# SIVsm Quasispecies Adaptation to a New Simian Host

**DOI:** 10.1371/journal.ppat.0010003

**Published:** 2005-09-30

**Authors:** Linda J Demma, John M Logsdon, Thomas H Vanderford, Mark B Feinberg, Silvija I Staprans

**Affiliations:** 1 Department of Biology, Emory University, Atlanta, Georgia, United States of America; 2 Departments of Medicine and Microbiology and Immunology, and Emory Vaccine Center, Emory University School of Medicine, Atlanta, Georgia, United States of America; National Institutes of Health, United States of America

## Abstract

Despite the potential for infectious agents harbored by other species to become emerging human pathogens, little is known about why some agents establish successful cross-species transmission, while others do not. The simian immunodeficiency viruses (SIVs), certain variants of which gave rise to the human HIV-1 and HIV-2 epidemics, have demonstrated tremendous success in infecting new host species, both simian and human. SIVsm from sooty mangabeys appears to have infected humans on several occasions, and was readily transmitted to nonnatural Asian macaque species, providing animal models of AIDS. Here we describe the first in-depth analysis of the tremendous SIVsm quasispecies sequence variation harbored by individual sooty mangabeys, and how this diverse quasispecies adapts to two different host species—new nonnatural rhesus macaque hosts and natural sooty mangabey hosts. Viral adaptation to rhesus macaques was associated with the immediate amplification of a phylogenetically related subset of *envelope (env)* variants. These variants contained a shorter variable region 1 loop and lacked two specific glycosylation sites, which may be selected for during acute infection. In contrast, transfer of SIVsm to its natural host did not subject the quasispecies to any significant selective pressures or bottleneck. After 100 d postinfection, variants more closely representative of the source inoculum reemerged in the macaques. This study describes an approach for elucidating how pathogens adapt to new host species, and highlights the particular importance of SIVsm *env* diversity in enabling cross-species transmission. The replicative advantage of a subset of SIVsm variants in macaques may be related to features of target cells or receptors that are specific to the new host environment, and may involve CD4-independent engagement of a viral coreceptor conserved among primates.

## Synopsis

Why do some infectious agents establish successful cross-species transmission while others do not? Despite the clear potential for diseases harbored by animals to become emerging human pathogens, this question remains unanswered. Certain simian immunodeficiency viruses (SIVs) responsible for the human HIV-1 and HIV-2 epidemics have succeeded in infecting new host species, including humans. This study provides clues to how an SIV adapts to a new host in an experimental cross-species transmission. Indeed, many emerging diseases are caused by highly mutation-prone RNA viruses like SIV, which exist not as a single species, but rather as a population of genetic variants within a single infection. The presence of numerous viral variants in an infected animal increases the chance that variants with the ability to enter into or multiply in a new host species are present. This study describes how an SIV population from a natural reservoir host, the sooty mangabey, adapts to a new monkey species, the rhesus macaque. A limited subset of SIV variants containing unique viral surface proteins appears well suited to multiply in the new host. This study documents how viral variation facilitates cross-species transmission, and highlights the particular importance of immunodeficiency virus envelope variants in infecting new hosts.

## Introduction

At least 40 primate species in Africa are infected by diverse simian immunodeficiency viruses (SIVs) assigned to six major phylogenetic lineages; however, the mosaic nature of the SIV genomes attests to the common simian-to-simian transmission of SIVs [1,2]. These African nonhuman primate reservoir hosts maintain normal CD4 T cell counts and avoid AIDS, despite lifelong SIV infection [3–6]. Our studies of naturally SIV-infected sooty mangabeys (SMs) indicate that these hosts are highly viremic, yet manifest far lower levels of aberrant immune activation and apoptosis than are seen in pathogenic SIV and HIV infections; these latter observations help to explain how SMs maintain numerically and functionally intact T lymphocyte populations [3]. Zoonotic transmission and sustained propagation of SIVcpz and SIVsm from SIV-infected chimpanzees and SMs, respectively, to humans [2,7], resulted in the human HIV-1 and HIV-2 AIDS epidemics.

SIV and HIV *env* sequence variation, including variation in length and glycosylation patterns, enables these viruses to utilize different coreceptors for infection, and to adapt to variation in the relative levels of the viral receptor (CD4) and coreceptors (e.g., CC-chemokine receptor 5 [CCR5]) to gain efficient entry into cells [8,9]. *Env* variation also enables the virus to readily escape antibody responses [10–12]. Our studies of SIVsm *env* diversity in naturally infected SMs demonstrate high levels of intrahost *env* variable region 1 and 2 (V1V2) amino acid diversity (median, 5.6%; range, 0%–38%) that are maintained by continual positive selection, presumably antibody mediated (unpublished data). Considerable V1V2 amino acid length variation and high and variable numbers of glycosylation consensus sequences are also observed. This high diversity of SIV V1V2 in the natural host environment may promote the potential for cross-species transmission by generating the *env* variants necessary to ensure successful infection of new hosts.

For successful cross-species transmission to occur, including the continued propagation of an infectious agent in a new host species, the agent must be able to replicate at levels in the new host that ensure its sustained passage to new individuals of that species; otherwise the newly infected host(s) will simply represent a “dead-end” infection that does not lead to secondary and sustained infections in the new species. Alternatively, the infectious agent that has been recently transmitted to a new host may require the accumulation of mutations that enable it to replicate at levels high enough to ensure continued transmission to new individuals. Thus, SIVs that are capable of quickly adapting to new hosts and replicating to high levels are most likely to successfully breach the species barrier and continue to spread in the new species. Adaptation of naturally occurring SIV quasispecies to new hosts has not been studied. In studies analyzing the adaptation of diverse HIV-1 quasispecies from identifiable human donors to newly infected “recipients,” the early expansion of viruses that are homogeneous in *env* sequences, macrophage-tropic, and CCR5-utilizing is described [13,14]. This sequence homogenization is not observed in *gag* [15], suggesting that multiple variants are transmitted, followed by selection for particular *env* variants during primary infection. Selection for *env* homogeneity has also been reported after parenteral inoculation [13], suggesting that, separate from any selective processes taking place at the mucosal barrier, there is strong selection for particular *env* genotypes during acute infection. Recently, a study of heterosexual HIV-1 transmission demonstrated that viruses encoding compact, glycan-restricted Envs with exposed neutralizing epitopes were significantly favored in newly infected hosts [16]. Another report confirmed these findings in transmission of subtype A but not B [17]. Whether these observations extend to other clades, cohorts, or routes of HIV infection remains to be determined [18,19].

Here we describe the adaptation of diverse SIVsm quasispecies to the new rhesus macaque (RM) host, and compare quasispecies evolution in natural SM and nonnatural RM hosts. During the first days of infection, SIVsm replicated as well in the RM host as in the original host, if not better, apparently due to the robust replicative capacity of a subset of viral variants containing a shorter V1 loop and lacking two specific glycosylation sites. This study demonstrates how viral quasispecies diversity, by providing multiple variants, some of which can replicate to high levels in new hosts, may facilitate cross-species transmission.

## Results

### High Diversity of the SIVsm Quasispecies Inoculum

The uncloned SIVsm inoculum consisting of plasma from a naturally infected SMs contained 4 × 10^6^ SIV RNA copies/ml. To characterize the diversity of this source inoculum (SI), and the molecular behavior of the quasispecies upon transmission to new hosts, we analyzed a 456-nucleotide region spanning the variable V1V2 region of *env* and a 421-nucleotide region of the p27 capsid region of the more functionally conserved *gag* gene (GenBank accession numbers AY852284–AY853166). We chose to sequence only portions of the coding sequences of these two genes, as efforts to amplify full-length coding sequences resulted in poor RT-PCR amplification efficiencies that were not compatible with the reliable sampling of multiple quasispecies variants. Sequences representing actively replicating SIV were amplified directly from virion RNA in the plasma by RT-PCR. 29 V1V2 and 7 *gag* clone sequences analyzed using maximum parsimony, neighbor-joining (NJ), and maximum likelihood (ML) phylogenetic tree constructions demonstrated that the SI was phylogenetically distinct from commonly used laboratory SIV isolates (Figure S1).

SI V1V2 sequence length varied between 139 and 143 aa (Table 1). The range of pairwise nucleotide diversity calculated for the SI population was 0.3%–5.1% for V1V2 (mean, 2.7%; median, 2.7%) and 0.7%-4.6% for *gag* (mean, 2.4%; median, 2.3%). The amino acid diversity ranged from 0% to 12.8% in V1V2 (mean, 5.9%; median, 6.3%) with only six of 406 identical pairwise comparisons. The amino acid diversity in *gag* p27 was lower, ranging from 0% to 0.7% (mean, 0.2%; median, 0%). The minimal diversity detected in *gag,* which was PCR-amplified using identical conditions, suggests that the observed V1V2 diversity is not the result of PCR-introduced mutation. The average viral diversity observed in this study is similar to that reported in other studies of SIV infections of natural hosts [4,20,21]. The within-host extremes of V1V2 diversity observed in this and another study (unpublished data) is a novel observation resulting from the large number of sequences analyzed.

**Table 1:**
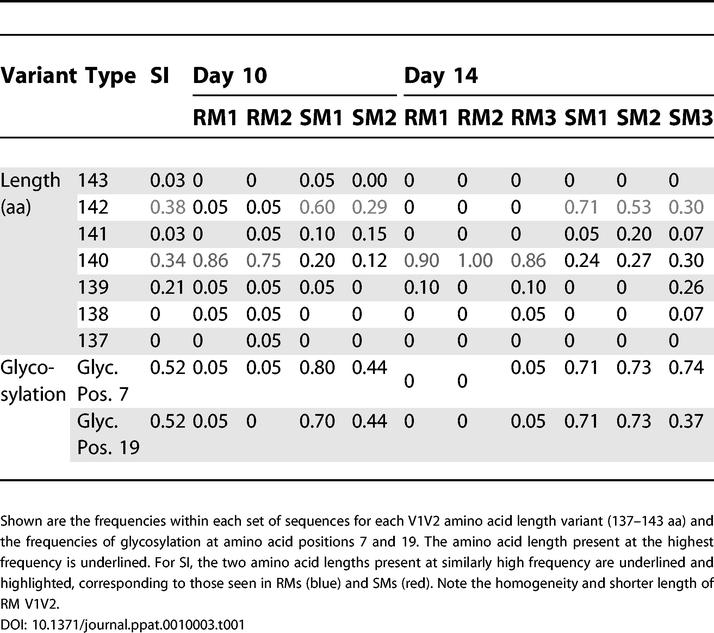
V1V2 Amnio Acid Variation in RMs and SMs at Days 10 and 14 and in SI

### Robust Virus Replication Demonstrates Immediate Quasispecies Adaptation to the New Nonnatural RM Host

SMs and RMs were intravenously (IV) inoculated with 1 ml of the SI described above (SIVsm). IV injection may partially recapitulate the circumstances of cross-species SIV transmission, which are thought to involve exposure to bloody flesh during hunting or butchering [22,23]. It ensures reproducible infection and enables the study of host-specific differences in response to SIV infection that lead to AIDS in RMs but not SMs [24]. At day 7 postinfection (p.i.), SIVsm replication was detected in all animals except RM2 (Figure S2). Peak viremia occurred between days 10 and 14 for all animals except RM2, whose peak likely occurred between days 14 and 28, an interval when no sampling was performed. RM1 and RM3 manifested peak viremia levels of 1.6 × 10^9^ copies/ml of plasma and 6.1 × 10^8^ copies/ml respectively, higher than the peak viremia for the three SMs (5.0 × 10^7^ to 1.5 × 10^8^ copies/ml). Viral loads declined to similar set point levels of ~1 × 10^6^ copies/ml, except for RM2, which maintained fewer than 1,000 copies/ml after day 60. RM1 and RM3 developed AIDS 2.5 and 3.5 y p.i. and were euthanized. Divergent host responses and disease outcomes during primary SIVsm infection of SMs and RMs are described elsewhere [24].

### Early Amplification of a Phylogenetically Related Subset of *env* Variants in RMs Contrasts with Unrestricted *env* Diversity in SMs

**Figure 1:**
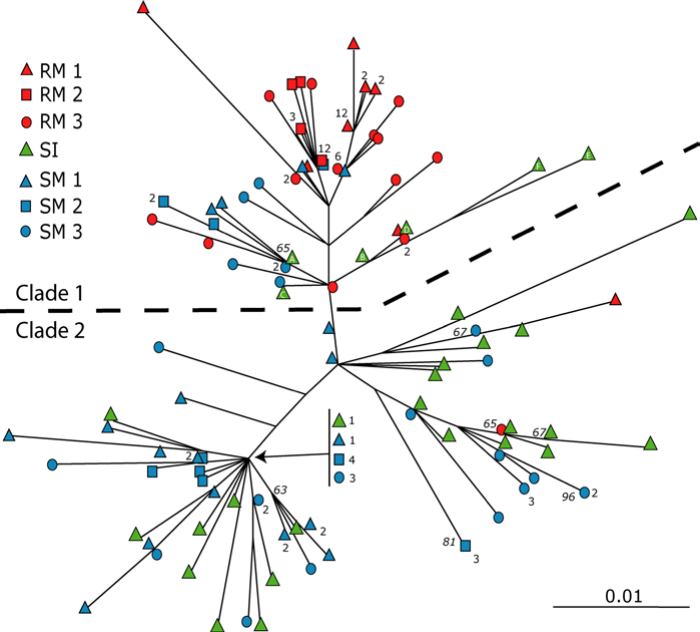
Phylogenetic Tree of V1V2 Variants An NJ tree of all day 14 and SI V1V2 variants, constructed using a GTR model of evolution. Bootstrap support values greater than 50% are shown in italics at nodes and the number of multiple clones from the same animal at the ends of branches is indicated beside the symbol.

At day 14 p.i., the replicating *env* V1V2 sequences for all six animals were compared to each other and to the SI. Despite the more robust replication of SIVsm in the RMs, few of the SI V1V2 variants appeared among the clade containing most of the variants replicating in RMs (clade 1; Figure 1), demonstrating that a subset of genetically related *env* variants was amplified during acute SIVsm infection of RMs. Specifically, the proportion of variants replicating in RMs in clade 1 (97%) was significantly higher than that of either the SI variants (21%; Marascuillo Procedure, *p* < 0.0001) or the variants replicating in SMs (22%; *p* < 0.0001). In contrast, there was little selection of specific SIVsm *env* variants upon transfer to new naïve SMs, with no significant difference (Marascuillo Procedure, *p* = 99%) in the distributions of SI variants and variants replicating in SMs between clades 1 and 2 (Figure 1). Mean intrahost pairwise nucleotide diversity in the RMs at day 14 was 0.9%, significantly lower than that of the SMs (2.1%; Tukey's HSD, *p* < 0.05) and the SI (Newman-Keuls, *p* < 0.05). Mean intrahost amino acid diversity in RMs at day 14 (1.6%) was lower than SM amino acid diversity (3.4%), although not significantly. This pattern of restriction was observed as early as 10 d p.i., with 37/39 (95%) of RM variants clustering with the same six SI variants seen at day 14 (unpublished data). Thus, only a subset of *env* genotypes appear well suited to replicate in the new RM host environment, but this subset replicates to surprisingly high levels.

### SIVsm *env* Variants That Are Preferentially Amplified in Newly Infected RMs Contain Shorter V1 Regions and Lack Two Glycosylation Consensus Sequences

**Figure 2:**
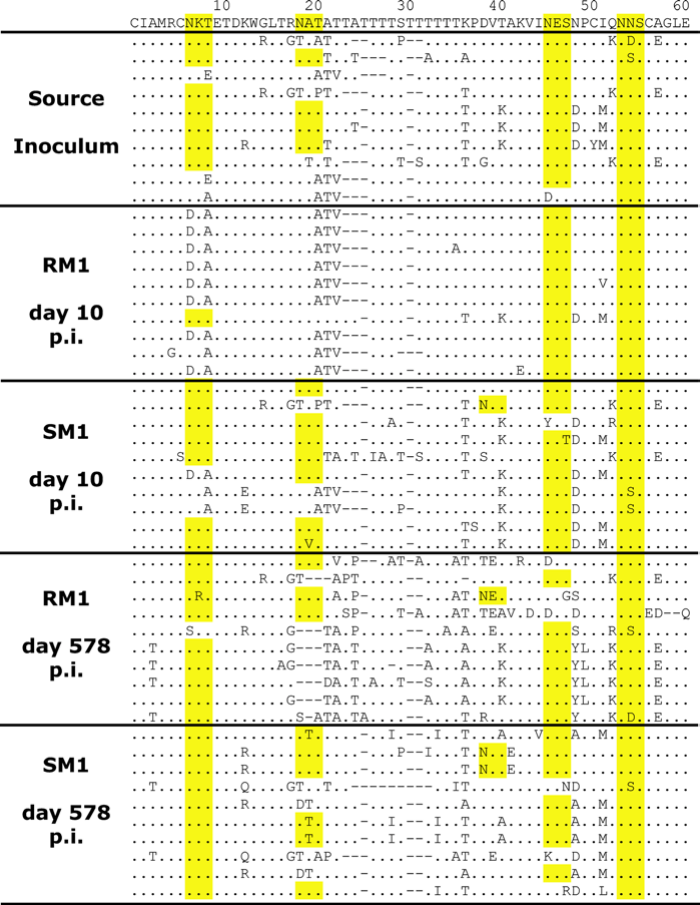
Pattern and Temporal Dynamics of Protein Sequence Evolution in Envelope V1V2 amino acid sequence for SI, SM1, and RM1 at days 10 and 578. The consensus of all sequences is indicated at the top with amino acid positions labeled above. Glycosylation consensus motifs (NXT/S) are highlighted in yellow.

SIV *env* glycosylation is important in receptor and coreceptor utilization [25], and in evading neutralizing antibodies [26,27]. Eight predicted N-linked glycosylation sites (N-gly) containing the amino acid motif NXT/S were identified along the 125 aa of V1V2 analyzed, although direct evidence of glycosylation at these sites is not explicitly demonstrated. Among the 29 SI clones analyzed, most of these positions encoded the consensus N-gly sequence (Figures 2 and S3), consistent with the observation that SIVsm V1V2 is highly glycosylated in its natural host (unpublished data).

Two of these predicted N-gly sites, at positions 7 and 19 in V1 (Figure 2), were immediately selected against in the newly infected RMs, before the anticipated development of an antibody response. (RMs and SMs demonstrated anti-SIV antibodies by ELISA at day 40 p.i., the first time-point assessed, with increasing titers by day 130 p.i. [unpublished data]). In the SI, 76% and 52% of V1V2 sequences exhibited the N-gly motif at positions 7 and 19. At 10 d p.i., the motif at position 7 was present in 80% of SM1 sequences, but in only 5% of RM1 sequences. At position 19, the motif was present in 70% of SM1 sequences compared to 5% of RM1 sequences (Figure 2 for SM1 and RM1 at day 10; Table 1 for frequencies in all animals). The near absence of the motif at positions 7 and 19 was observed in all RMs analyzed at days 10 and 14 (Figure S3). Furthermore, the predominant V1V2 variants in RMs at days 10 and 14 were shorter in length by two amino acids compared to the variants in SM (Table 1). Thus, a disadvantage of variants with longer V1 loops and two specific N-gly sites in V1 may explain the restricted outgrowth of specific *env* variants during early infection of RMs.

At days 10–100, SIVsm sequences from SMs had a greater mean number of N-gly sites per sequence than variants in RMs, but by day 578 the overall frequency of glycosylation consensus motifs increased in both species, and there was no difference between species (Figure S4; *p* < 0.001). This increase in mean glycosylation over time in the RMs is in part due to the reemergence of variants containing the two specific N-gly sites that were absent in the majority of early RM variants. The range of V1 region amino acid length variations also increased over time, and no species-specific differences were seen at day 100 and thereafter (unpublished data). These data demonstrate continual evolution of V1 in both SMs and RMs.

### Increasing Positive Selection in SMs and RMs at Later Times Postinfection

**Figure 3:**
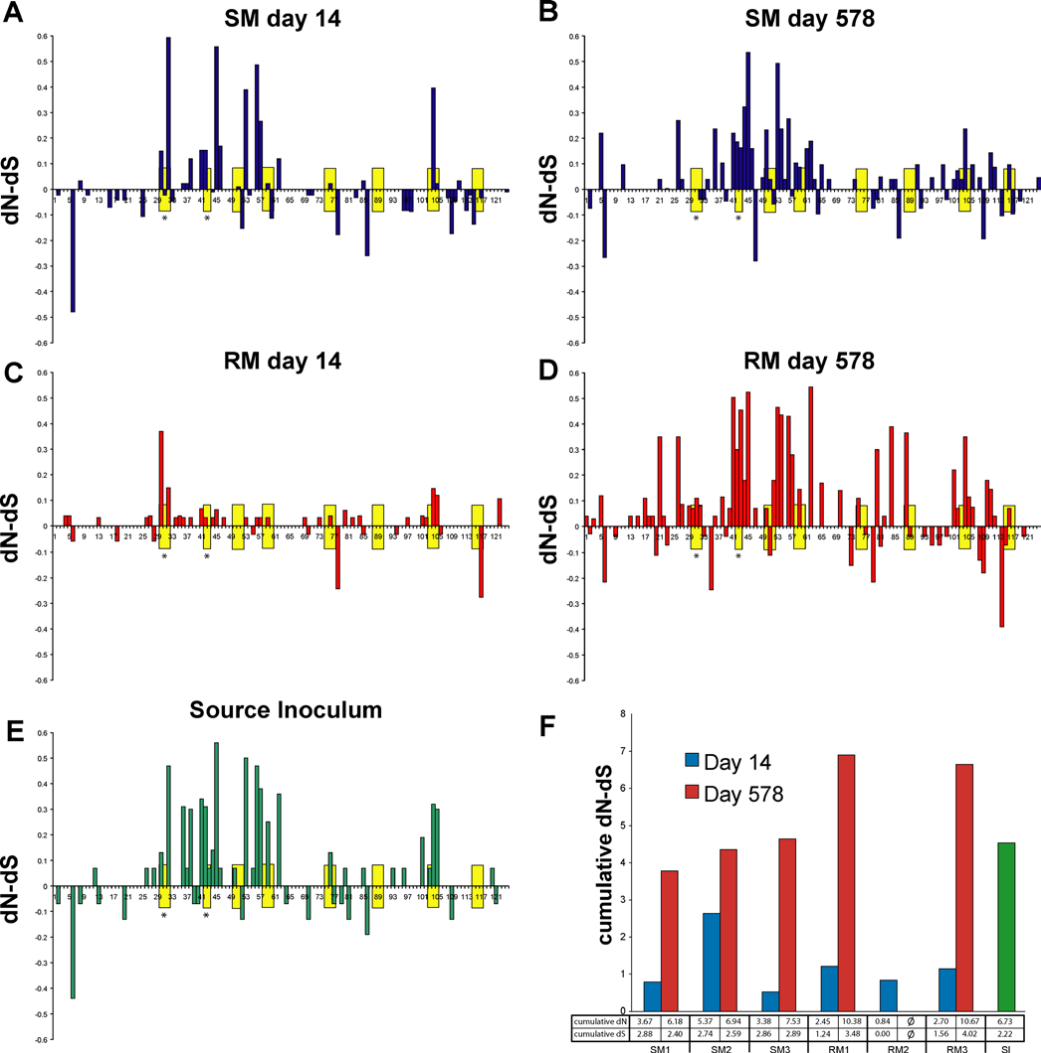
Evidence for Greater Positive Selection in SIV *env* at Later Times Postinfection (A–E) Calculations for dN and dS were performed along the 124 amino acids of the V1V2 region using SNAP (http://hiv-web.lanl.gov/). The average dN and dS at each codon is shown for SMs at day 14 (A) and day 578 (B), as well as for RMs at day 14 (C) and day 578 (D), and for the SI (E). Yellow boxes indicate predicted N-gly sites, and asterisks indicate N-gly sites not present at early time points in RMs. (F) Cumulative dN and dS are shown across all sites for each animal at day 14 and day 578. Raw values of cumulative dN and cumulative dS are indicated below the graph.

To compare selection pressures between hosts and time points, nonsynonymous and synonymous nucleotide substitutions (dN and dS, respectively) at each codon of V1V2 were calculated for sequences at day 14 (prior to seroconversion) and day 578 (chronic infection) for each animal and the SI (Figure 3). The pattern of selection in SMs at both time points (Figure 3A and 3B) was similar to the SI (Figure 3E), suggesting few changes in selection pressure upon SIVsm transfer to naïve SMs. This is consistent with the phylogenetic analyses, which indicate that IV transfer of SIVsm does not subject the quasispecies to any significant selective pressures or bottleneck in the natural SM host, but results in considerable restriction of the SIVsm quasispecies diversity in RMs. In contrast to the SMs, the relative lack of sites under strong selection in RMs at day 14 (Figure 3C) corroborates the strong, early selection of a subset of variants from the SI. The subsequent substantial increase (Figure 3D) in the number of sites under selection and the magnitude of selection at those sites not only reflect the outgrowth of variants more similar to the SI, but also suggest the presence of immune-selective pressures in the RMs during the postacute phase of infection.

To quantify the magnitude of selection in the SIVsm *env* V1V2, cumulative dN and cumulative dS were calculated for each animal at days 14 and 578 (Figure 3F). SMs and RMs showed relative increases in cumulative dN and dS at day 578 (Wilcoxon rank sum test, *p* < 0.005), especially in a region of V1 (amino acid positions 22–57) described as important in antibody escape [25,28–30]. At later times, despite increases of both dN and dS in RMs, cumulative dN-dS was greater in RMs than SMs, although the difference was not statistically significant, suggesting greater positive selection pressures in the non-natural host. However, continual evolution of V1V2 occurred in both species, consistent with observations of persistent within-host positive diversifying selection in SMs (unpublished data).

### Variants Related to the Original Inoculum Reemerge in RMs at Later Times Postinfection

**Figure 4:**
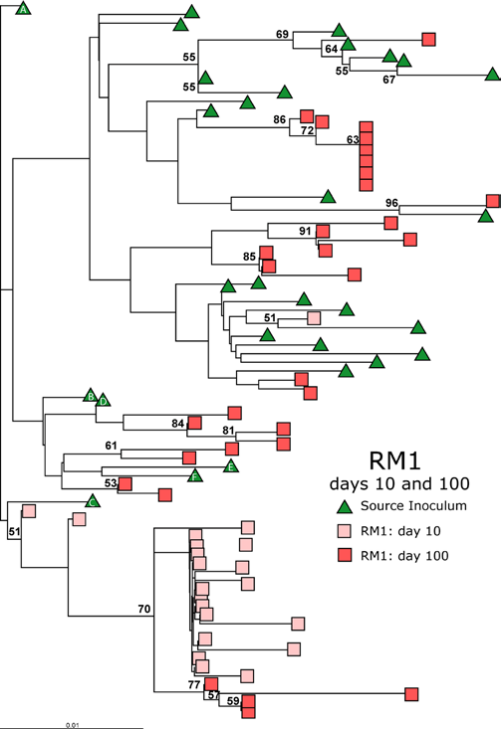
Reemergence of Donor-Related Variants in Late Infection of Nonnatural Hosts ML tree of sequences from the SI and RM1 at days 10 and 100. Bootstrap values greater than 50% are shown at nodes. The SI variants are identified by the legend.

Phylogenetic analyses of clones from day 100 p.i. showed V1V2 sequences beginning to diversify in RMs, although the variants remained clustered by host (Figure 4; see Figure S5 for parallel phylogenetic analysis of SM1). At this time, viral sequences in RM1 were more closely related to variants from the SI than to variants from 10 d p.i., suggesting a reemergence of the SI-related quasispecies during chronic infection. These results indicate that day 10 V1V2 variants are an evolutionary “dead-end,” as it is unlikely that directional evolution would result in viral quasispecies in all RMs that are highly related to the original SI quasispecies.

**Figure 5:**
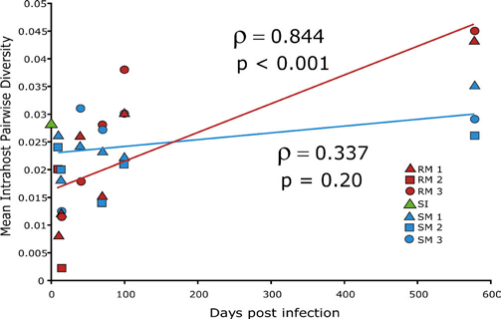
Temporal Changes in Nucleotide Sequence Evolution in SMs and RMs Mean pairwise nucleotide diversity of the V1V2 sequences for each animal at each time point, calculated using the Tamura-Nei model of nucleotide substitution in MEGA 2.1 [52]. The diversity of the SI is indicated on the y-axis. Trend lines are drawn for RMs (red) and SMs (blue).

At all time points after infection of the three SMs, average nucleotide diversity of SIVsm V1V2 sequences remained similar to that of the original SI (~3.0%; Figure 5). In contrast, in RMs, nucleotide diversity increased after day 40 despite manifesting an initial restriction in viral diversity. Viral variants at day 578 became more animal-specific (unpublished data), as would be expected under host-specific selection pressure. The viral diversity in RMs at day 578 (averaging 4.5% ± 0.8%) was greater than both that of the SI and that of the SIVsm variants observed in SMs at late times (t-test with Bonferroni adjustment, *p* = 0.03). These data suggest that selection pressures change during the course of SIVsm infection of RMs; V1V2 variants that replicated to high levels in primary infection lost their replicative advantage, and previously undetected variants that were closely related to the SI became detectable. The increasing nucleotide diversity over time in RMs is consistent with the observed increase in positive diversifying selection pressures in RMs (see Figure 3).

### No Early Selection for Specific *gag* Variants Following Intra- or Cross-Species Transmission

**Figure 6:**
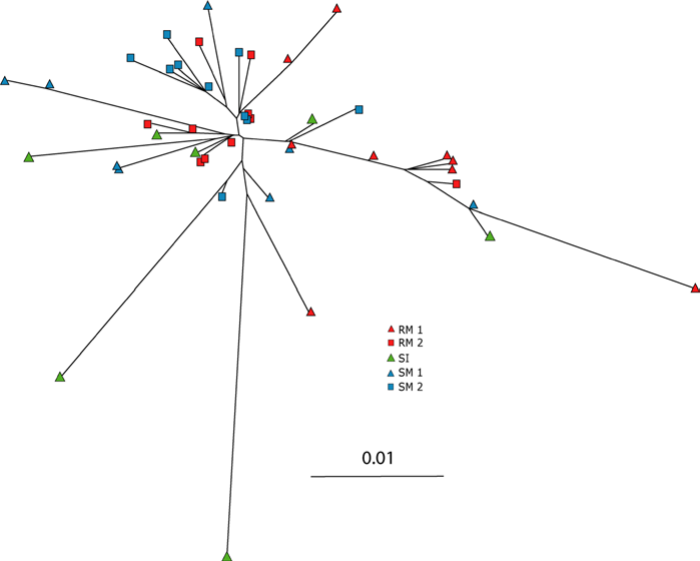
Phylogenetic Analysis of SIVsmm *gag* Variants in Natural and Nonnatural Hosts An NJ unrooted phylogenetic tree of all day-10 *gag* variants was constructed in PAUP* [43] using the GTR model with a gamma rate distribution of shape α = 1.0. The SI variants are represented by triangles and identified by the legend. Bootstrap values greater than 50% are shown at nodes, and the number of multiple clones from the same animal at the ends of branches is indicated within the symbol.

Despite the high levels of selection in *env,* no species-specific phylogenetic relationships were observed for SIVsm *gag* variants at day 10 p.i. (Figure 6), indicating that there was no preferential amplification of specific *gag* variants in association with the establishment of infection in either SMs or RMs. The average nucleotide diversity of *gag* variants following transmission to both species was similar to that of the SI (unpublished data). These data suggest that most *gag* variants were equivalent in their ability to establish successful infection of either host. At later times, some amino acid changes in *gag* became apparent in individual animals (Figure S6). In both RMs analyzed at days 100 and 578, there was almost complete amino acid fixation at two sites (positions 39 and 68). Only one SM manifested any evidence of amino acid fixation in *gag,* and this was only partial (position 126 in SM2). Fixation of amino acid changes, particularly at position 68, which occurred in two RMs, could be due to cell-mediated immune response pressures, which are thought to be stronger in RMs compared to SMs [24]. However, that these changes are due to random amino acid fixation through genetic drift cannot be ruled out, because of the large population sizes involved and the limited number of *gag* clones analyzed.

## Discussion

Identification of specific characteristics that enable pathogens to infect new species may reveal why some emerging infections become widespread while others do not. RNA virus quasispecies diversity has been posited to underlie their zoonotic success, yet no study had analyzed the behavior of diverse naturally occurring viral quasispecies upon inoculation into different host species. This study represents the first analysis, to our knowledge, of the evolution of a diverse naturally occurring SIV quasispecies, following its side-by-side inoculation into a new nonnatural host species (rhesus macaques) and the natural host species from which it was derived (sooty mangabeys). Our studies, which focused on the intensive sequencing of large numbers of viral variants in the *env* V1V2 region, point to the importance of diversity in this region in initiating a successful cross-species infection event. However, this does not exclude the possibility that diversity in other genome regions, including diversity in other *env* regions, plays an important role in cross-species transmission events.

Upon inoculation of SMs with the diverse SIVsm quasispecies, little host restriction was observed during acute infection despite continued strong positive selection pressures consistent with host-specific viral evolution and similar to our findings in a study of natural infection in SMs (unpublished data). However, a restricted, genetically related subset of SIVsm *env* V1V2 variants that harbored a shorter V1 loop and lacked two specific glycosylation sites was preferentially amplified in all of the RMs during acute infection. This was observed despite IV inoculation, which would have bypassed mucosal barriers, and was observed as early as 10 days p.i., likely prior to the development of immune responses. While we cannot rule out that these variants hitchhiked to a high frequency in the RMs, the observed amplification of a subset of variants appeared to represent an advantage for these envelopes that was related to specific features of target cells in the RM but not the SM. Loss of one of these glycosylation sites (position 7) has been shown to result in CD4-independent SIVs in the SIVmac239 strain [25], suggesting the possibility that viral variants that preferentially use CCR5 independently of CD4 may be selected for during acute infection of the new RM host. It remains to be determined whether loss of this same glycosylation site in SIVsm also results in CD4 independence. Because CCR5 is more highly conserved between SMs and RMs than is CD4 [31,32], it might be anticipated that CD4-independent viral variants could overcome species differences in the primary viral receptor (CD4) and have a distinct advantage in the new host environment. The possibility that efficient coreceptor utilization independent of CD4 is an important factor in establishing cross-species transmission is a topic for further study.

Recently, the selection of more compact, glycan-restricted HIV *envs* after heterosexual transmission was described [16,17]. If the selection of slightly shorter, glycan-restricted SIVsm *envs* observed in this study of cross-species transmission is a related phenomenon, then, because our IV inoculations bypassed mucosal barriers, the advantage of such variants may be a posttransmission phenomenon. One possibility is that in an antibody-naïve host environment, more compact, less glycosylated Env conformations with more accessible receptor-binding domains have a replicative advantage. However, it is noteworthy that SIVsms encoding less glycosylated V1V2 regions do not appear to have any replicative advantage in newly infected, antibody-naïve SMs. The lack of selective pressure on the SIVsm quasispecies in acutely infected SMs suggests that highly glycosylated V1V2 variants are well adapted to initiate new infections of its natural host species.

Although it might be expected that only a subset of SM-adapted SIVs could replicate well in a new species, it is intriguing that these variants replicated to levels exceeding those seen in the natural SM host. In this [24] and other studies of acute SIV infection [33,34], we have observed a relationship between the magnitude of early CD4 T cell activation and the magnitude of early virus replication. Given the higher levels of CD4 T cell activation observed in the acutely infected RMs as compared to the SMs in this study [24], it is conceivable that increased numbers of activated CD4 T cells provided additional cellular targets for infection. If this target cell-driven hypothesis of more robust SIVsm replication in RMs is correct, it raises the possibility that SIV infection-induced CD4 T cell activation in nonnatural hosts actually facilitates zoonotic transmission of these CD4 T cell-tropic lentiviruses. Additional studies are required to explore this hypothesis. It is also worth noting that activated CD4 T cells may up-regulate CCR5 (or other coreceptor) expression [35], and down-regulate CD4 expression [36]. This might provide another selective force for the observed glycan-restricted SIVsm variants that may be less CD4 dependent in newly infected RMs. Whatever the explanation for the selection of specific *env* V1V2 variants in RMs, selection pressures giving rise to these effects need not be strong, given the high level of diversity of the inoculum and the likely number of replication cycles involved. Nonetheless, the robust replication of these variants ensured the establishment of high viremia during infection of a new host, a characteristic that would be important for continued propagation of the virus in the new species.

At later times p.i. of RMs, SIVsm variants more closely related to the original SI quasispecies reemerged, suggesting that all variants were initially transmitted to the RMs, but that only a subset of variants replicated to high levels during the acute infection period. Variants related to the SI may have been physically sequestered in resting memory cells, as has been suggested for HIV-1 [37], or simply replicated at such low levels that they were not sampled. Studies have suggested that “archival” HIV variants are maintained in infected hosts [38]. When host selection pressures change, such as with the termination of antiretroviral therapy, these archived variants may emerge, obviating the necessity for back mutation of the most predominant viral variants at the time of change in selection pressure. This capacity to archive the variants present in a diverse, infecting swarm, referred to as the “molecular memory” of the quasispecies [39], demonstrates the significant potential of lentiviral quasispecies to respond to changing selection pressures and presents significant hurdles when considering HIV prevention or treatment measures. SI V1V2 variant emergence at later times suggests that these viruses have replicative advantages in chronically infected RMs, perhaps due to their resistance to neutralizing antibodies. Compensatory changes in other regions of the genome (e.g., in the CD4-binding region of SIV env) could also have relieved initial selection pressures against these variants.

This study demonstrates how viral quasispecies diversity enables successful cross-species transmission by providing multiple variants, some of which are able to establish high-level viremia in new hosts, which, in turn, increases the probability of successful propagation within new species. Our studies point to SIVsm *env* diversity in its reservoir host as a likely required, although not necessarily sufficient prerequisite for successful cross-species transmission. These observations have implications for which infectious agents may be zoonotically transmitted and efficiently propagated in a new host species. Finally, the potential roles of CD4-independent SIVs and coreceptor sequence conservation in cross-species transmission are important topics for further study.

## Materials and Methods

### Experimental SIV infection.

SMs and RMs were housed at the Yerkes National Primate Research Center, Atlanta, Georgia, United States, and maintained in accordance with federal guidelines [40]. Prior to the study, the absence of SIV infection was confirmed by negative SIV PCR of plasma and negative HIV-2 serology for at least 1 y. Three RMs and three SMs were experimentally infected IV with a diverse inoculum of uncloned SIVsm from a naturally infected SM (individual FQi). SMs FLn, FCo, and FGu are referred to as SM1, SM2, and SM3, respectively. RMs RHt4, RQl4, and RZw4 are referred to as RM1, RM2, and RM3, respectively. The animals were followed at multiple time points following the infection, and quantitative PCR was carried out to determine the viral dynamics of their acute SIV infection [24].

### PCR.

Viral RNA was extracted from freshly thawed plasma samples from the three SMs and three RMs in this study using the Qiagen Viral RNA Kit. SIV sequences were amplified from 5 μL of template in a PCR reaction using the Qiagen One-Step RT-PCR Kit (Qiagen, Valencia, California, United States).

To amplify the *env* V1V2 region, a mixture of two forward primers was used, FENV1 (5′-CTTGGGAGAATACAGTCACAG-3′) corresponding to bp 6,780–6,800 of the SIVsmmH4 genome, and FENV2 (5′-CTTGGGAGAATACAGTAACAG-3′) also corresponding to bp 6,780–6,800 but containing one different base at position 6,796. The reverse *env* V1V2 primer was also a mixture of RENV1 (5′-TAAATCTAATAGCATCCCAATAAT-3′) and RENV2 (5′-TAAATCTAATAGCATCCCAATAGT-3′) corresponding to bp 7,221–7,244 of the SIVsmmH4 genome, and differing at bp 7,222. The primer pair amplified a 456-bp fragments spanning the V1V2 hypervariable region of *env*.

The *gag* region was amplified using shortgagF1 (5′-TTAAGTCCAAGAACATTAAATGC-3′) and shortgagR (5′-GTAGAACCTGTCTACATAGCT-3′), which correspond to bp 1,493–1,515 and 1,937–1,957 of SIVsmmH4, respectively, yielding a 421-bp product of the 5′ end of the p27 capsid protein.

Conditions for each reaction were 30 min at 50 °C and 15 min at 95 °C, followed by 40 cycles of 94 °C for 1 min, 52 °C for 30 s, and 72 °C for 1 min. A final extension time was carried out for 5 min at 72 °C. Due to extremely low viral load, RNA from RM2 could not be amplified after day 14 for either V1V2 or *gag*. RT-PCR sensitivity was determined to be less than 500 copies per reaction. Viral loads from each of the animals did not significantly differ at each time point (with the exception of animal RM3, in which virus was undetectable using the RT-PCR protocol after day 14). Samples were not standardized for input copy number, potentially confounding the extent of change in viral diversity that was measured over time, although this would not confound comparisons between animals at each time point since viral loads were similar.

No-template controls and negative controls from the RNA extraction were used in each set of reactions to ensure that no cross contamination occurred at either step. In addition, samples from each pair of animals, SM1/RM1, SM2/RM2, SM3/RM3 were extracted at least 3 mo apart. This ensured that contamination within species was avoided. Contamination of negative-extraction controls was detected when extracting SM2 and RM2 samples from days 70 and 100. This extraction was repeated, and virus could not be amplified from RM2 due to very low copy numbers. On one occasion, the RT-PCR reaction was contaminated with a particular molecular clone, however these sequences were easy to identify with phylogenetic analysis due to their extensive divergence from the SI. These contaminants were excluded from the analysis. RNA extracted from day 10 plasma in SM1 and RM1 was RT-PCR amplified under the same conditions as above, except that 10 μL of PCR product was removed at 25, 30, 35, and 40 cycles for cloning and sequencing to ensure that PCR bias during extended cycling was not a factor in sample diversity. Viral RNA from days 70 and 100 for RM1 and RM3 was extracted, PCR amplified, and cloned in duplicate to ensure experimental repeatability. A 1:10 dilution of SI was amplified under the same conditions, and 15 clones from this RT-PCR product were sequenced to ensure that input copy number did not bias diversity.

### DNA cloning and sequencing.

PCR products from each sample were run on a 1.5% low-melt agarose gel, and the 456-bp V1V2 or 421-bp *gag* product was extracted and cloned into the pCR4-TOPO vector (TOPO TA Cloning Kit, Invitrogen, Carlsbad, California, United States). Between 15 and 30 V1V2 clones and 5 and 10 *gag* p27 clones from each time point and each animal were randomly selected and sequenced using the M13F and M13R primers with the dye terminator cycle sequencing method.

### Sequence and phylogenetic analyses.

Sequences were aligned using the program CLUSTAL X [41], followed by manual adjustment using MacClade 4.0 [42]. Nonaligned regions of length variation in V1 and V2 were removed (corresponding to nucleotides 6,932–6,974), and sequences containing internal stop codons, single deletions, or double deletions were also excluded from analysis, as these are thought to be PCR artifacts [43]. Figures S1 and S2 show the resulting alignment of all sequences in V1V2 and *gag,* respectively.

For the SI, maximum parsimony and NJ were implemented using the PAUP 4.0b10* package for V1V2 and *gag* [44]. For each of the resulting trees, bootstrap support was determined with 1,000 resamplings of the sequences. The most highly supported clade in both the NJ and the parsimony trees was used as the outgroup for all subsequent phylogenetic trees (Figure S3).

For tree construction, the Modeltest program [45] was used to construct and evaluate the DNA substitution models used. Based on the Modeltest results, phylogenetic analysis on sequences obtained from successive time points during the acute infection was performed by ML using the program Treefinder [46]. The general time reversible (GTR) model, which allows for rate variation between sites [47–49], was used, and the shape parameter (α) of the gamma distribution used in this model was estimated, as were base frequencies and substitution rate parameters. Bootstrap support was determined with 1,000 resamplings of the ML tree using distance methods in PAUP4.0b10*, incorporating the estimated rate parameters.

The cumulative number of synonymous and nonsynonymous and nucleotide substitutions was estimated using Synonymous/Nonsynonymous Analysis (SNAP; http://hiv-web.lanl.gov/), which calculates rates of nucleotide substitution from a set of codon-aligned nucleotide sequences, based on the method of Nei and Gojobori [50], and incorporating a statistic developed in Ota and Nei [51]. Viral nucleotide diversity at each time point was determined by calculating the pairwise nucleic acid distances for each of the clones using the method of Tamura and Nei [52] in the program MEGA 2.1 [53]. This same method was also employed to quantify nucleotide divergence from the source, defined as the ratio of the difference in nucleotide diversity between SI and each sample of variants to the total diversity in the two groups. Amino acid diversity was calculated using the gamma distance method in the program Mega 2.1. Phylogenetic trees constructed with synonymous or nonsynonymous sites only were constructed in Mega 2.1 using distance methods incorporating the Tamura-Nei model of nucleotide substitution with gamma-distributed rates. All statistics were computed using SYSTAT 10 software (SPSS, Chicago, Illinois, United States).

## Supporting Information

Figure S1 Phylogenetic Analysis of Source Inoculum V1V2 Variants with Molecular Clones (A) NJ tree showing the most highly supported clade of SI used as the outgroup for all subsequent phylogenetic trees. (B) An unrooted ML tree of 30 SI V1V2 variants and corresponding V1V2 sequences from clones SIVmac239, SIVsmmH4, and SIVmne (obtained from the HIV sequence database [http://hiv-web.lanl.gov/content/index]) was constructed with Treefinder [46] using a GTR model and estimated gamma rate distribution, base frequencies, and substitution rates. Bootstrap values greater than 50% are shown at nodes.
(53 KB PDF)

Figure S2 Viral Replication Dynamics following Infection with a Diverse SIVsmm Three SMs and three RMs were inoculated with plasma obtained from a naturally infected SM. Viral replication was monitored in SMs and RMs by quantitative RT-PCR of plasma RNA (see Materials and Methods).
(210 KB PDF)

Figure S3 Amino Acid Sequences of All Animals and All Time Points in the V1V2 Region of Envelope Region corresponds to nucleotides 6,801–7,220 of SIVsmmH4. Sequences were aligned using the program CLUSTAL X [41], followed by manual adjustment using MacClade 4.0 [42]. A nonaligned region of length variation in V1 was removed, corresponding to amino acids 129–137 of SIVsmmH4 *env,* and is indicated by “**~**”. The consensus of all sequences in this study is shown above all sample sets, with codon positions labeled above. A dot indicates amino acid identity with the consensus sequence, and any amino acid changes are indicated with the appropriate symbol. The V1V2 regions are highlighted in blue on the consensus sequence, and glycosylation consensus motifs present in each sequence are highlighted in yellow.
(226 KB DOC)

Figure S4 Mean Number of Glycosylation Consensus Motifs in SMs and RMs for All Time Points Frequency of glycosylation consensus motifs is lower in RMs (regression analysis, *p* < 0.001) and increases over time in both SMs and RMs. The number of motifs in the SI is indicated with a star on the y-axis.
(114 KB PDF)

Figure S5 Phylogenetic Analysis of Natural Host V1V2 Variants at Days 10–100 Shows No Specific Pattern ML phylogenetic tree of sequences obtained from SM1 at days 10 (pink) to 100 (red) is shown, constructed with Treefinder [46] using a GTR model and estimated gamma rate distribution, base frequencies, and substitution rates. Bootstrap values greater than 50% are shown at nodes, and the number of multiple clones from the same animal at the ends of branches is indicated within the symbol. The SI variants are represented by triangles and identified by the label within.
(77 KB PDF)

Figure S6 Amino Acid Sequences of All Animals and All Time Points in the p27 Region of *gag*
Region corresponds to nucleotides 1,516–1,936 of SIVsmmH4. Sequences were aligned using the program CLUSTAL X [41], followed by manual adjustment using MacClade 4.0 [42]. The top sequence in each set corresponds to the majority consensus sequence from all sequences at all time points, with codon positions labeled above. A dot indicates amino acid identity with the consensus sequence, and any amino acid changes are indicated with the appropriate symbol.
(75 KB DOC)

### Accession Number

The GenBank (http://www.ncbi.nlm.nih.gov/) accession number of the SIVsmmH4 genome is X14307.

## Abbreviations

CCR5: CC-chemokine receptor 5
GTR: general time reversible
IV: intravenous
NJ: neighbor-joining
ML: maximum likelihood
p.i.: postinfection
RM: rhesus macaque
SI: source inoculum
SIV: simian immunodeficiency virus
SM: sooty mangabey
V1: SIV *envelope* variable region 1
V2: SIV *envelope* variable region 2
