# Supplementary figures and images for: SIVsm Quasispecies Adaptation to a New Simian Host
Source: PLoS Pathog. 2005 Sep 30;1(1):e3. doi: 10.1371/journal.ppat.0010003 (PMC1238738; doi:10.1371/journal.ppat.0010003)

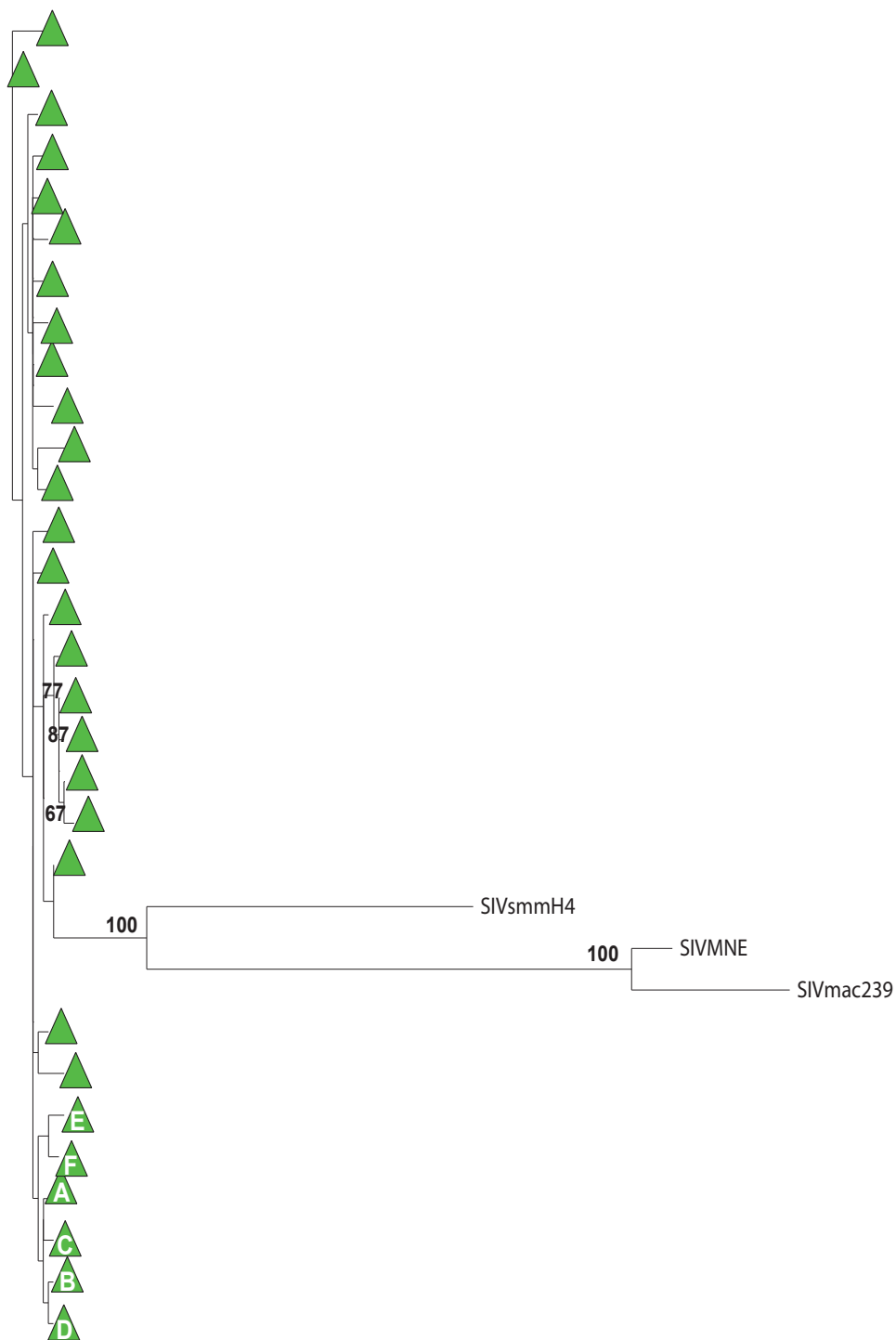

0.1 substitutions/site

Supplement: Figure S1 — (A) NJ tree showing the most highly supported clade of SI used as the outgroup for all subsequent phylogenetic trees. (B) An unrooted ML tree of 30 SI V1V2 variants and corresponding V1V2 sequences from clones SIVmac239, SIVsmmH4, and SIVmne (obtained from the HIV sequence database [http://hiv-web.lanl.gov/content/index]) was constructed with Treefinder [46] using a GTR model and estimated gamma rate distribution, base frequencies, and substitution rates. Bootstrap values greater than 50% are shown at nodes. (53 KB PDF) [file ppat.0010003.sg001.pdf]

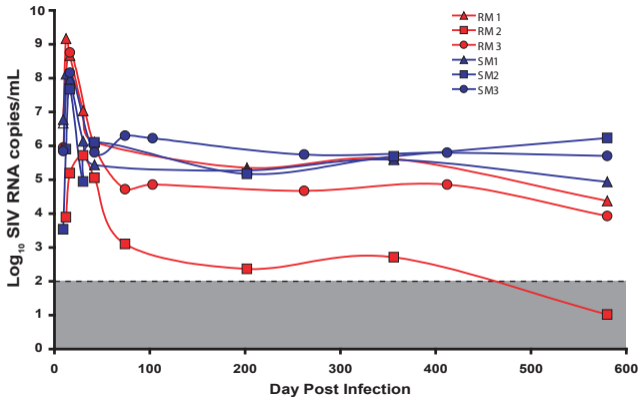

Supplement: Figure S2 — Three SMs and three RMs were inoculated with plasma obtained from a naturally infected SM. Viral replication was monitored in SMs and RMs by quantitative RT-PCR of plasma RNA (see Materials and Methods). (210 KB PDF) [file ppat.0010003.sg002.pdf]

Mean No. Glyc. Sites Per Sample

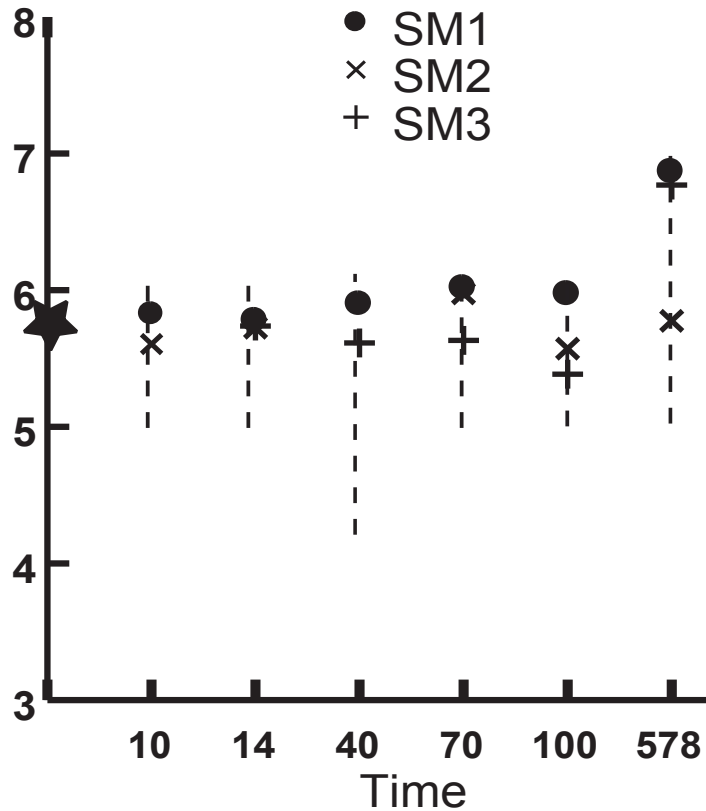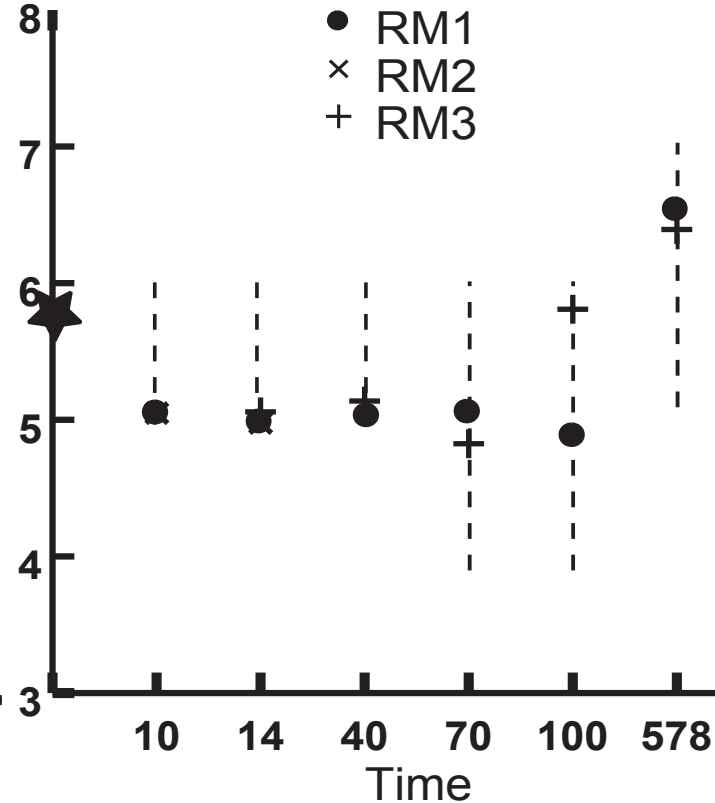

Supplement: Figure S4 — Frequency of glycosylation consensus motifs is lower in RMs (regression analysis, p < 0.001) and increases over time in both SMs and RMs. The number of motifs in the SI is indicated with a star on the y-axis. (114 KB PDF) [file ppat.0010003.sg004.pdf]

# SM 1: Days 10-100

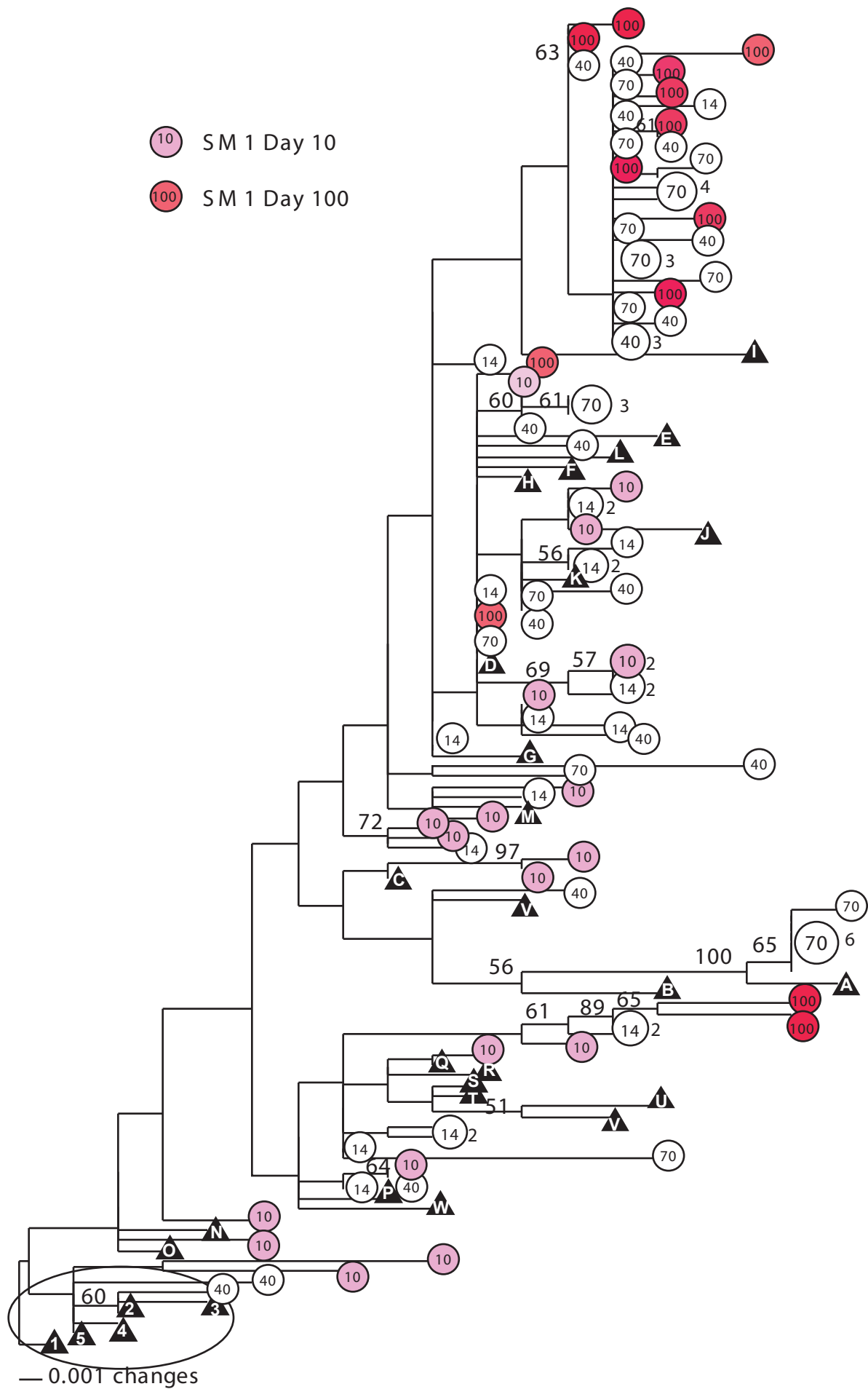

Supplement: Figure S5 — ML phylogenetic tree of sequences obtained from SM1 at days 10 (pink) to 100 (red) is shown, constructed with Treefinder [46] using a GTR model and estimated gamma rate distribution, base frequencies, and substitution rates. Bootstrap values greater than 50% are shown at nodes, and the number of multiple clones from the same animal at the ends of branches is indicated within the symbol. The SI variants are represented by triangles and identified by the label within. (77 KB PDF) [file ppat.0010003.sg005.pdf]
