# Supplementary material for: SIVsm Quasispecies Adaptation to a New Simian Host
Source: PLoS Pathog. 2005 Sep 30;1(1):e3. doi: 10.1371/journal.ppat.0010003 (PMC1238738; doi:10.1371/journal.ppat.0010003)
Supplement: Figure S3 — Region corresponds to nucleotides 6,801–7,220 of SIVsmmH4. Sequences were aligned using the program CLUSTAL X [41], followed by manual adjustment using MacClade 4.0 [42]. A nonaligned region of length variation in V1 was removed, corresponding to amino acids 129–137 of SIVsmmH4 env, and is indicated by “~”. The consensus of all sequences in this study is shown above all sample sets, with codon positions labeled above. A dot indicates amino acid identity with the consensus sequence, and any amino acid changes are indicated with the appropriate symbol. The V1V2 regions are highlighted in blue on the consensus sequence, and glycosylation consensus motifs present in each sequence are highlighted in yellow. (226 KB DOC) [file ppat.0010003.sg003.doc]

**SI**

10 20 30 40 50 60 70 80 90 100 110 120

QAIEDVWNLFETSIKPCVKLTPLCIAMRCNKTETDKWGLTRNAT~~TTPDVTAKVINESNPCIQNNSCAGLEQEPMVSCKFNMTGLKRDKKKEYNETWYSRDLVCEQSTNESESKCYMNHCNTSVIQESCD

FQiv1 .........................T.........QR...GT..~~.....K.......D.....S..V..............................................................

FQiv2 ...................................Q........~~.....K.......D.....S.................................................................

FQiv3 ...............................A...R.......A~~.K...........................................................G..KN...................

FQiv4 ............................................~~.....K.......D..M....................................................................

FQiv5 .....................................R..GT.A~~.K...............K.D..E..............................................................

FQiv6 .............................S.A...........A~~.K..............................................................GN...................

FQiv7 ............................................~~.K..I...................................................I............................

FQiv8 .............................S.....R....GT..~~AA..E..............S.................................................................

FQiv9 ............................................~~.....K.......D..M...................I...R............................................

FQiv10 .....................................R...T.P~~...G.............K....E..............................................................

FQiv11 ............................................~~...S.................................................................................

FQiv12 ............................................~~.....K.......D..M....................................................................

FQiv13 ............................................~~.A.................S................I................................................

FQiv14 ...............................E...........A~~.K..............................................................G....................

FQiv15 ............................................~~.....K.......D..M....................................................................

FQiv16 .....................................R..GT.P~~.................K....E..............................................................

FQiv17 ...........I................................~~.....K.......D..M............................R.................D.....................

FQiv18 ............................................~~.K...A.......D..M....................................................................

FQiv19 ............................................~~.....K.......D..M.........................G..........P...............................

FQiv20 .....................................R..GT.P~~.................K....E..............................................................

FQiv21 ............................................~~.....K.......D..M............................................G...N...................

FQiv22 .....................................R..GT.P~~.................K....E..............................................................

FQiv23 ...............................A...........A~~.K..............................................................GN...................

FQiv24 ...................................R........~~.....K.......D.YM....................................................................

FQiv25 ............................................~~...S..E..........K....E..............................................................

FQiv26 ..........................................T.~~...G.............K....E..............................................................

FQiv27 ...............................E...........A~~.K...........................................................G...N...................

FQiv28 ...........................S................~~.K...K.......D..M...............................................................R....

FQiv29 ...............................A...........A~~.K........D..........................................................................

**SM1 Day 10**

10 20 30 40 50 60 70 80 90 100 110 120

QAIEDVWNLFETSIKPCVKLTPLCIAMRCNKTETDKWGLTRNAT~~TTPDVTAKVINESNPCIQNNSCAGLEQEPMVSCKFNMTGLKRDKKKEYNETWYSRDLVCEQSTNESESKCYMNHCNTSVIQESCD

FLn10v1 ............................................~~.K..............................................................G....................

FLn10v2 .....................................R..GT.P~~...N.............K....E..............................................................

FLn10v3 ............................................~~....IK.......D...R...................................................................

FLn10v4 .....................................R..GT.P~~.................K....E..............................................................

FLn10v5 ............................................~~.K...K.......D..M....................................................................

FLn10v6 ............................................~~.K...K.......D..M....................................................................

FLn10v7 ........P...................................~~.....K....Y..D...R...................................................................

FLn10v8 ............................................~~.....K......TD..M....................................................................

FLn10v9 ............................S...............~~...S.............K....E.........................................GN...................

FLn10v10 ............................................~~...N.........D...K....E........................................................T.....

FLn10v11 ............................................~~.....K.......D..M...............................................G....................

FLn10v12 ............................................~~...N..E..............................................................................

FLn10v13 ............................................~~..S..K.......D..M....................................................................

FLn10v14 .............................D.A............~~.....K.......D..M....................................................................

FLn10v15 ...............................A...E.......A~~.K...K.......D.....S.................................................................

FLn10v16 ...............................A...E.......A~~.K...K.......D.....S.................................................................

FLn10v17 ............................................~~..S..K..........D..M.................................................................

FLn10v18 ............................................~~.....K..........D..M.................................................................

FLn10v19 ..........................................V.~~.....K.......D..M....................................................................

FLn10v20 ...............................E...........A~~.K..............................................................G....................

**SM2 Day 10**

10 20 30 40 50 60 70 80 90 100 110 120

QAIEDVWNLFETSIKPCVKLTPLCIAMRCNKTETDKWGLTRNAT~~TTPDVTAKVINESNPCIQNNSCAGLEQEPMVSCKFNMTGLKRDKKKEYNETWYSRDLVCEQSTNESESKCYMNHCNTSVIQESCD

FCo10v1 ...............................E...........A~~.K..............................................................G....................

FCo10v2 ...............................E...........A~~.K..............................................................G....................

FCo10v3 ............................................~~.K............S.....................V..................E.............................

FCo10v4 ............................................~~...S..E..........K...................................................................

FCo10v5 ...............................E...........A~~.K..............................................................G....................

FCo10v6 ............................................~~.....K.......D..M....................................................................

FCo10v7 ............................................~~..L..K.......D..M...............................................G....................

FCo10v8 .....A......................................~~...S..E..........K.............................................D.....................

FCo10v9 ..............................N.............~~.....K.......D..M..........................................................H..A......

FCo10v10 ............................................~~...S..E..........K...................................................................

FCo10v11 ............................................~~.K............S.....................V......................................S.........

FCo10v12 ............................................~~.K............S.....................V................................................

FCo10v13 ............................................~~...S..E..........K...................................................................

FCo10v14 ............................................~~..L..K.......D..M...............................................G....................

FCo10v15 ...............................E...........A~~.K..............................................................G....................

FCo10v16 ............................................~~..L..K.......D..M...............................................G....................

FCo10v17 ............................................~~...S..E..........K...................................................................

FCo10v18 ............................................~~.....K.......D..M....................................................................

FCo10v19 ............................................~~.....K.......D..M....................................................................

**RM1 Day 10**

10 20 30 40 50 60 70 80 90 100 110 120

QAIEDVWNLFETSIKPCVKLTPLCIAMRCNKTETDKWGLTRNAT~~TTPDVTAKVINESNPCIQNNSCAGLEQEPMVSCKFNMTGLKRDKKKEYNETWYSRDLVCEQSTNESESKCYMNHCNTSVIQESCD

RHt10v1 .............................D.A...........A~~.K..............................................................G.................F..

RHt10v2 .............................D.A...........A~~.K..............................................................G.................F..

RHt10v3 .............................D.A...........A~~.K..............................................................G.................F..

RHt10v4 .............................D.A...........A~~.K..................................I.........................A.G.................F..

RHt10v5 .............................D.A...........A~~AK..............................................................G.................F..

RHt10v6 .............................D.A...........A~~.K..............................................................G.................F..

RHt10v7 .............................D.A...........A~~.K..............................................................G.................F..

RHt10v8 ....................A........D.A...........A~~.K..............................................................G.................F..

RHt10v9 .............................D.A...........A~~.K...................................................................................

RHt10v10 .............................D.A...........A~~.K..............V...............................................G.................F..

RHt10v11 .............................D.A...........A~~.K..............................................................G.................F..

RHt10v12 ............................................~~.....K.......D..M....................................................................

RHt10v13 .............................D.A...........A~~.K..............................................................G.................F..

RHt10v14 .............................D.A...........A~~.K..............................................................G.................F..

RHt10v15 .............................D.A...........A~~.K..............................................................G.................F..

RHt10v16 .............................D.A...........A~~.K..............................................................G.................F..

RHt10v17 .............................D.A...........A~~.K..............................................................G.................F..

RHt10v18 .............................D.A...........A~~.K..............................................................G.................F..

RHt10v19 ...........................G...A...........A~~-K...............................T..............................G.................F..

RHt10v20 .............................D.A...........A~~.K...................................................................................

RHt10v21 .............................D.A...........A~~.K.....E........................................................G.................F..

**RM2 Day 10**

10 20 30 40 50 60 70 80 90 100 110 120

QAIEDVWNLFETSIKPCVKLTPLCIAMRCNKTETDKWGLTRNAT~~TTPDVTAKVINESNPCIQNNSCAGLEQEPMVSCKFNMTGLKRDKKKEYNETWYSRDLVCEQSTNESESKCYMNHCNTSVIQESCD

RQl10v1 ...............................E...........A~~KK......................................E.......................GN...................

RQl10v2 .............................D.A...........A~~.K..............................................................G....................

RQl10v3 .............................D.A...........A~~.K..............................................................G....................

RQl10v4 .............................D.A...........A~~.K..............................................................G....................

RQl10v5 .............................D.A...........A~~.K..............................................................G....................

RQl10v7 ...............................A............~~AA..E..............S..........................................I......................

RQl10v8 ...............................E...E.......A~~KK......................................E.......................GN.......R...........

RQl10v9 ...........................................-~~.....K.......D..M....................................................................

RQl10v10 ...............................E...........A~~.K........D..D..M.......................N.......................GN...................

RQl10v11 .............................D.A...........A~~.K..............................................................G.......S............

RQl10v12 .............................D.A...........A~~.K..............................................................G....................

RQl10v13 .............................D.A...........A~~.K..............................................................G....................

RQl10v14 ...............................E...........A~~.K........D..D..M.......................N.......................GN.....V.............

RQl10v15 .............................D.A...........A~~.K..............................................................G....................

RQl10v16 .............................D.A.A.........A~~.K..............................................................G....................

RQl10v17 ...............................E...........A~~.K.....................D................N.......................GN...................

RQl10v18 .............................D.A.A.........A~~.K..............................................................G....................

RQl10v19 .............................D.A.A.........A~~.K..............................................................G....................

RQl10v20 ...............................E...........A~~.K......................................N.G.....................G....................

RQl10v21 .............................D.A...........A~~.K..............................................................G....................

**SM1 Day 14**

10 20 30 40 50 60 70 80 90 100 110 120

QAIEDVWNLFETSIKPCVKLTPLCIAMRCNKTETDKWGLTRNAT~~TTPDVTAKVINESNPCIQNNSCAGLEQEPMVSCKFNMTGLKRDKKKEYNETWYSRDLVCEQSTNESESKCYMNHCNTSVIQESCD

FLn14v1 ............................................~~.....K.......D..M...............................................G....................

FLn14v2. ...............................E...........A~~.K...................................................................................

FLn14v3. ............................................~~.K...K.......D..M....................................................................

FLn14v5. ........................T...................~~.....K.......D..M....................................................................

FLn14v6. ............................................~~.K...K.......D..M....................................................................

FLn14v7. ............................................~~...S..E..............................................................................

FLn14v8. ............................................~~..S..K.......D..M....................................................................

FLn14v9. ............................................~~.....K.......D..M....................................................................

FLn14v10 ............................................~~.....K.......D..M............................E.......................................

FLn14v11 ...............................E...........A~~.K...................................................................................

FLn14v12 ............................................~~.....R.......D..M...............................................G....................

FLn14v13. ............................................~~.K...K.......D..M....................................................................

FLn14v14. ............................................~~..S..K.......D..M....................................................................

FLn14v15. .......S....................................~~.K...K.......D..M....................................................................

FLn14v16. ...............................A...........A~~.K...................................................................................

FLn14v17. ............................................~~.....A.......D..M...................................................................G

FLn14v18. ............................................~~.....K.......D..M....................................................................

FLn14v19. ............................................~~.....K.....G.D...R.....................................................V.............

FLn14v20. .............................D.A...........A~~.K..............................................................G....................

FLn14v21. .............................D.A...........A~~.K..............................................................G....................

**SM2 Day 14**

10 20 30 40 50 60 70 80 90 100 110 120

QAIEDVWNLFETSIKPCVKLTPLCIAMRCNKTETDKWGLTRNAT~~TTPDVTAKVINESNPCIQNNSCAGLEQEPMVSCKFNMTGLKRDKKKEYNETWYSRDLVCEQSTNESESKCYMNHCNTSVIQESCD

FCo14v1 .............................D.A...........A~~.K..............................................................G....................

FCo14v2 ............................................~~.....K.......D..M....................................................................

FCo14v3 ............................................~~.....K.......D..M...............................................G....................

FCo14v4 ............................................~~.....K.......D..M...............................................G....................

FCo14v5 ............................................~~.....K.......D..M....................................................................

FCo14v6 ...............................E...........A~~.K..............................................................G....................

FCo14v7 ...............................E...........A~~.K..............................................................G....................

FCo14v8 ...............................E...........A~~.K..............................................................G....................

FCo14v9 ............................................~~.....K.......D..M....................................................................

FCo14v10 ............................................~~.....K.......D..M....................................................................

FCo14v11 ............................................~~.....K.......D..M....................................................................

FCo14v12 ............................................~~...S..E..........K...................................................................

FCo14v13 ............................................~~.....K.......D..M....................................................................

FCo14v14 ............................................~~...S..E..........K...................................................................

FCo14v15 ............................................~~...S..E..........K...................................................................

**SM3 Day 14**

10 20 30 40 50 60 70 80 90 100 110 120

QAIEDVWNLFETSIKPCVKLTPLCIAMRCNKTETDKWGLTRNAT~~TTPDVTAKVINESNPCIQNNSCAGLEQEPMVSCKFNMTGLKRDKKKEYNETWYSRDLVCEQSTNESESKCYMNHCNTSVIQESCD

FGu14v1 .............................S.....R....GT..~~AA..E..............S.................................................................

FGu14v2 .....................................R..GT.P~~.....K...........K....E.........................................G....................

FGu14v3 ............................................~~.....K.......D..M....................................................................

FGu14v4 .....................................R..GT.P~~.....K.......D..M...............................................GN...................

FGu14v5 ...............................E...........A~~.K..............................................................G....................

FGu14v6 ............................................~~.K...K.......D..M....................................................................

FGu14v7 .....................................R..GT.P~~.....K...........K....E.........................................G....................

FGu14v8 ............................................~~.....K.......D..M...................V................................................

FGu14v9 ...............................E...........A~~.K..............................................................G....................

FGu14v10 ............................................~~.K...K.......D..M....................................................................

FGu14v11 ...................................R........~~.K...................................................................................

FGu14v12 ............................................~~.....K.......D..M....................................................................

FGu14v13 ............................................~~...N.............K....E..............................................................

FGu14v14 .............................S.............A~~.K...................................................................................

FGu14v15 ............................................~~...S.............K....E..............................................................

FGu14v16 .....................................R..GT..~~.....K...........K....E.........................................G....................

FGu14v17 .....................................R..GT.P~~.................K....E..............................................................

FGu14v18 .....................................R..GT.P~~.....K...........K....E.........................................G....................

FGu14v19 ............................................~~.....K.......D..M....................................................................

FGu14v20 ............................................~~.....K.......D..M....................................................................

FGu14v21 ...........................................A~~.K..............................................................G....................

FGu14v22 ............................................ ~~.....K.......D..M....................................................................

FGu14v23 .....................................R..GT.P~~.................K....E..............................................................

FGu14v24 ...............................A...........A~~.K....V.........................................................G....................

FGu14v25 ...............................A....R......A~~.K..............................................................G....................

FGu14v26 .....................................R..GT.P~~.................K....E..............................................................

FGu14v27 ...............................A...........A~~.K..............................................................G....................

**RM1 Day 14**

10 20 30 40 50 60 70 80 90 100 110 120

QAIEDVWNLFETSIKPCVKLTPLCIAMRCNKTETDKWGLTRNAT~~TTPDVTAKVINESNPCIQNNSCAGLEQEPMVSCKFNMTGLKRDKKKEYNETWYSRDLVCEQSTNESESKCYMNHCNTSVIQESCD

RHt14v1 .............................D.A...........A~~.K..............................................................G.................F..

RHt14v2 .............................D.A...........A~~.K..............................................................G.................F..

RHt14v3 .............................D.A...........A~~.K...............................T..............................G.................F..

RHt14v4 .............................D.A...........A~~.K..............................................................G.................F..

RHt14v5 .........................T...S.....R....GT..~~AA..EA.E...........S......................N..........................................

RHt14v6 .............................D.A...........A~~.K..............................................................G.................F..

RHt14v7 .............................S.A...........A~~.K..............................................................GN...................

RHt14v8 .............................D.A...........A~~.K..............................................................G....................

RHt14v9 .............................D.A...........A~~.K..............................................................G.................F..

RHt14v10 .............................D.A...........A~~.K..............................................................G.................F..

RHt14v11 .............................D.A...........A~~.K..............................................................G.................F..

RHt14v12 .............................D.A...........A~~.K..............................................................G.................F..

RHt14v13 .............................D.A...........A~~.K..............................................................G.................F..

RHt14v14 .............................D.A...........A~~.....K.......D..M............V.......................................................

RHt14v15 .............................D.A...........A~~.K..............................................................G.................F..

RHt14v16 .............................D.A...........A~~.K..............................................................G.................F..

RHt14v17 .............................D.A...........A~~.K..............................................................G.................F..

RHt14v18 .............................D.A...........A~~.K..............................................................G.................F..

RHt14v19 .............................D.A...........A~~.K..............................................................G.................F..

RHt14v20 .............................D.A...........A~~.K..............................................................G.................F..

RHt14v21 .............................D.A...........A~~.K..............................................................G.................F..

**RM2 Day 14**

10 20 30 40 50 60 70 80 90 100 110 120

QAIEDVWNLFETSIKPCVKLTPLCIAMRCNKTETDKWGLTRNAT~~TTPDVTAKVINESNPCIQNNSCAGLEQEPMVSCKFNMTGLKRDKKKEYNETWYSRDLVCEQSTNESESKCYMNHCNTSVIQESCD

RQl14v1 .............................D.A...........A~~.K..............................................................G....................

RQl14v2 .............................D.A...........A~~.K...........................................................G..G....................

RQl14v3 .............................D.A..G........A~~.K..............................................................G....................

RQl14v4 .............................D.A...........A~~.K..............................................................G....................

RQl14v5 .............................D.A...........A~~.K..........................................R...................G....................

RQl14v6 .............................D.A...........A~~.K..............................................................G....................

RQl14v7 .............................D.A...........A~~.K..............................................................G....................

RQl14v8 .............................D.A...........A~~.K..............................................................G....................

RQl14v9 .............................D.A...........A~~.K..............................................................G....................

RQl14v10 .............................D.A...........A~~.K..............................................................G....................

RQl14v11 .............................D.A...........A~~.K..............................................................G....................

RQl14v12 .............................D.A...........A~~.K..............................................................G....................

RQl14v13 .............................D.A...........A~~.K..............................................................G....................

RQl14v14 .............................D.A...........A~~.K..............................................................G....................

RQl14v15 ...DY........................D.A...........A~~.K..............................................................G....................

RQl14v16 ..........................V..D.A...........A~~.K..................................V...........................G....................

RQl14v17 .............................D.A...........A~~.K..............................................................G....................

**RM3 Day 14**

10 20 30 40 50 60 70 80 90 100 110 120

QAIEDVWNLFETSIKPCVKLTPLCIAMRCNKTETDKWGLTRNAT~~TTPDVTAKVINESNPCIQNNSCAGLEQEPMVSCKFNMTGLKRDKKKEYNETWYSRDLVCEQSTNESESKCYMNHCNTSVIQESCD

RZw14v1 ...............................E...........A~~.K......................................N.......................G....................

RZw14v2 .............................D.A...........A~~.K..............................................................G....................

RZw14v3 .............................D.A...........A~~.K...................................................................................

RZw14v4 .............................D.A...........A~~.K..............................................................G....................

RZw14v5 .............................D.A...........A~~.K..............................................................G....................

RZw14v6 .............................S.A...........A~~.K..............................................................G....................

RZw14v7 .............................D.A...........A~~.K..............................................................G....................

RZw14v8 ...............................E...........A~~.K......................................N.......................GN...................

RZw14v9 .............................D.A...........A~~.K...................................................................................

RZw14v10 .............................D.A.A.........A~~.K..............................................................G....................

RZw14v11 .....................................R..GT.A~~.K...............K....E..............................................................

RZw14v12 .............................D.A...........A~~.K..............................................................G....................

RZw14v13 .............................D.A...........A~~.K..............................................................G....................

RZw14v14 .............................D.A...........A~~.K............................................................A.G....................

RZw14v15 .............................D.A...........A~~.K..............................................................G....................

RZw14v16 .............................S.A...........A~~.K..............................................................GN...................

RZw14v17 .............................S.A...........A~~.K..............................................................GN...................

RZw14v18 ...............................A...........A~~.K..............................................................G....................

RZw14v19 ...............................E...........A~~.K.......................................................I......G....................

RZw14v20 ............P................D.A...........A~~.K..............................................................G....................

RZw14v21 .............................D.A...........A~~.K..............................................................G....................

**SM1 Day 40**

10 20 30 40 50 60 70 80 90 100 110 120

QAIEDVWNLFETSIKPCVKLTPLCIAMRCNKTETDKWGLTRNAT~~TTPDVTAKVINESNPCIQNNSCAGLEQEPMVSCKFNMTGLKRDKKKEYNETWYSRDLVCEQSTNESESKCYMNHCNTSVIQESCD

FLn40v2 ............................................~~...N..E..............................................................................

FLn40v3 ......................................S.....~~.....K.......D..M....................................................................

FLn40v4 ............................................~~.....A.......D..M....................................................................

FLn40v5 ............................................~~.....K.......D..M....................................................................

FLn40v6 ............................................~~.....A.......D..M....................................................................

FLn40v7 ............................................~~.....A.......D..M....................................................................

FLn40v8 .........................T.........Q....GT..~~.....K.......D.....S.................................................................

FLn40v9 ................................D...........~~.....K.......D..M.........................A..........................................

FLn40v11 .....................S......................~~.K...K.......D..M..............................................................T.....

FLn40v12 ............................................~~.....K.......D..M....................................................................

FLn40v13 ............................................~~.K...K.......D..M...............................................G....................

FLn40v14 ............................................~~.-...A.......D..M....................................................................

FLn40v15 ..........................................V.~~.....K.......D..M....................................................................

FLn40v16 ...............................I............~~.-.G.A.......D..M............I.......................................................

FLn40v17 .....................................R..GT.A~~.................K....E......................R.......................................

FLn40v18 ............................................~~.....A.......D..M....................................................................

FLn40v19 ............................................~~.A..E....V.........S.................................................................

FLn40v20 ............................................~~.....A.......D..M....................................................................

FLn40v21 ............................................~~.....A.......D..M....................................................................

FLn40v22 ............................................~~.....A.......D..M....................................................................

FLn40v23 ............................................~~.....A.......D..M....................................................................

FLn40v24 ............................................~~.....A......ND..M....................................................................

FLn40v25 ............................................~~.K...K.......D..M....................................................................

FLn40v26 ............................................~~.....K.......D..M....................................................................

FLn40v27 ............................................~~..S..K.......D..M....................................................................

FLn40v28 .........................T.........Q....GT..~~.....K.......D.....S.................................................................

FLn40v29 ...............................A...........A~~.K........D.......................L.............................G....................

FLn40v30 ............................................~~...N..E..............................................................................

**SM3 Day 40**

10 20 30 40 50 60 70 80 90 100 110 120

QAIEDVWNLFETSIKPCVKLTPLCIAMRCNKTETDKWGLTRNAT~~TTPDVTAKVINESNPCIQNNSCAGLEQEPMVSCKFNMTGLKRDKKKEYNETWYSRDLVCEQSTNESESKCYMNHCNTSVIQESCD

FGu40v1 .............................S.A...........A~~.K.............................................ND...............GN...................

FGu40v2 .............................S.A...........A~~.K.............................................ND...............GN...................

FGu40v3 ........P............................R..GT.P~~.................K....E..............................................................

FGu40v4 .......................Y.....S.....R....GT..~~.A..E........S...R.S.................................................................

FGu40v5 .....................................R..GT.P~~.................K....E..............................................................

FGu40v6 ...............................A...........A~~.K....................E....................................D.........................

FGu40v7 .....................................R..GT.P~~...............R.....................................................................

FGu40v9 ...................................R........~~.....K.......D..M....................................................................

FGu40v10 ...................................R........~~.....K.......D..M..............G.....................................................

FGu40v11 .....................................R..GT.P~~.................K....E..............................................................

FGu40v12 ...................................R..S.....~~.....K.......D..M....................................................................

FGu40v13 ...............................E...........A~~.K..............................................................GN...................

FGu40v14 .....................................R..GT.P~~.................K....E.....................................................I........

FGu40v15 ............................................~~...S.............K....E..G.....G................................G....................

FGu40v16 ...................................R........~~.....K.......D..M................................................G...................

FGu40v17 .....................................R..GT.P~~.................K....E..............................................................

FGu40v18 ............................................~~...S.............K....E..............................................................

FGu40v19 ...............................A...........A~~.K..............................................................GN...................

FGu40v20 .....................................R..GT.P~~.................K....E.....................R........................................

FGu40v21 ...............................E...........A~~.K..............................................................G....................

FGu40v22 ............................................~~.....K.......D..M....................................................................

**RM1 Day 40**

10 20 30 40 50 60 70 80 90 100 110 120

QAIEDVWNLFETSIKPCVKLTPLCIAMRCNKTETDKWGLTRNAT~~TTPDVTAKVINESNPCIQNNSCAGLEQEPMVSCKFNMTGLKRDKKKEYNETWYSRDLVCEQSTNESESKCYMNHCNTSVIQESCD

RHt40v1 .........................T.....A........GT..~~A...E........S...R.S.................................................................

RHt40v2 ...................S...........E...........A~~KK......................................E.......................GN...................

RHt40v3 .........................T...S.....R....GT..~~AA..EA.E...........S.................................................................

RHt40v4 ...............................E...........A~~KK......................................E.......................GN...................

RHt40v5 ...............................A............~~.....K.......D..M...............................................GN...................

RHt40v6 ...............................A...........A~~.K...............................T...................................................

RHt40v7 .........................T.....A........GT.A~~A...E............R............................................A......................

RHt40v8 .........................T.....A........GT..~~A...E........S...R.S.................................................................

RHt40v9 .T.......................T.....A........GT..~~A...E........S...R.S.....................K...........................................

RHt40v10 ...............................A............~~.....K.......D..M...............................................GN...................

RHt40v11 ...............................A...........A~~.K...............................T...................................................

RHt40v12 .........................T.....A........GT..~~A...E........S...R.S.................................................................

RHt40v13 .........................T.....A........GT.A~~A...E............R...................................................................

RHt40v14 ...............................A...........A~~.K...............................T...................................................

RHt40v15 ...............................E...........A~~KK......................................E.......................GN...................

RHt40v16 ...G.....................T.....A........GT.A~~A...EI...........R....V.................E............................................

RHt40v17 ...............................E...........A~~KK......................................E.......................GN...................

RHt40v18 ...............................A...........A~~.K...............................T...................................................

RHt40v19 ...............................E...........A~~.K......................................N.G........-............G....................

RHt40v20 ...G.....................T.....A........GT.A~~A...EI...........R....V.................E............................................

**RM3 Day 40**

10 20 30 40 50 60 70 80 90 100 110 120

QAIEDVWNLFETSIKPCVKLTPLCIAMRCNKTETDKWGLTRNAT~~TTPDVTAKVINESNPCIQNNSCAGLEQEPMVSCKFNMTGLKRDKKKEYNETWYSRDLVCEQSTNESESKCYMNHCNTSVIQESCD

RZw40v1 .............................S.A...........A~~.K..............................................................GN...................

RZw40v2 .............................S.A...........A~~.K..............................................................GN...................

RZw40v3 .............................S.A...........A~~.K......................................N.......................GN...................

RZw40v4 .............................S.A...........A~~.K..............................................................GN...................

RZw40v5 .............................S.A...........A~~.K..............................................................GN...................

RZw40v6 ............................................~~..S..K.......D..M....................................................................

RZw40v7 .............................S.A...........A~~.K..............................................................GN...................

RZw40v8 .............................S.A...........A~~.K..............................................................GN...................

RZw40v9 ...............................A...........A~~.K....V...........D.............................................G....................

RZw40v10 ............................................~~...N..E.........................................................GN...................

RZw40v11 .............................S.A...........A~~.K..............................................................GN...................

RZw40v12 ...............................E...........A~~.K..............................................................G....................

RZw40v13 ......................................S.....~~.K...K.......D..M...........................................R........................

RZw40v14 .............................S.AK..........A~~.K..............................................................GN...................

RZw40v15 ............................................~~...S..E..........K...................................................................

RZw40v23 .............................S.A...........A~~.K..............................................................GN...................

RZw40v29 .............................S.A...........A~~.K..............................................................GN...................

**SM1 Day 70**

10 20 30 40 50 60 70 80 90 100 110 120

QAIEDVWNLFETSIKPCVKLTPLCIAMRCNKTETDKWGLTRNAT~~TTPDVTAKVINESNPCIQNNSCAGLEQEPMVSCKFNMTGLKRDKKKEYNETWYSRDLVCEQSTNESESKCYMNHCNTSVIQESCD

FLn70v5 .........................T.........Q....GT..~~.....K.......D.....S.................................................................

FLn70v6 ............................................~~.....A.......D..M....................................................................

FLn70v7 ............................................~~.....A.......D..M....................................................................

FLn70v9 ............................................~~.....K.......D..M....................................................................

FLn70v10 ............................................~~.....A.......D..M...............................................G....................

FLn70v11 ............................................~~.....A.......D..M....................................................................

FLn70v12 ............................................~~.....A.......D..M...............................................G....................

FLn70v13 ............................................~~..S..K.......D..M....................................................................

FLn70v14 .........................T.........Q....GT..~~.....K.......G.....S.................................................................

FLn70v15 .................G..........................~~.....A.......D..M....................................................................

FLn70v16 ............................................~~.....K.......D..M....................................................................

FLn70v17 ...............................A...........A~~.K....V..............................................................................

FLn70v18 ............................................~~.....A.......D..M....................................................................

FLn70v19 ..........................................V.~~.....K.......D..M.......................................S............................

FLn70v20 .........................T.........Q....GT..~~.....K.......D.....S.................................................................

FLn70v21 ..........................................V.~~.....K.......D..M.......................................S............................

FLn70v22 ............................................~~.....A.......D..M....................................................................

FLn70v23 ............................................~~.....A.......D..M....................................................................

FLn70v24 .........................T.........Q....GT..~~.....K.......D.....S.................................................................

FLn70v25 ..........................................V.~~.....K.......D..M.......................................S............................

FLn70v26 .........................T.........Q....GT..~~.....K.......D.....S.................................................................

FLn70v27 ..........................................V.~~.....K.......D..M.......................................S............................

FLn70v28 .........................T.........Q....GT..~~.....K.......D.....S.................................................................

FLn70v29 ...............................A...........A~~.....A.......D..M....................................................................

FLn70v30 .........................T.........Q....GT..~~.....K.......D.....S.................................................................

**SM2 Day 70**

10 20 30 40 50 60 70 80 90 100 110 120

QAIEDVWNLFETSIKPCVKLTPLCIAMRCNKTETDKWGLTRNAT~~TTPDVTAKVINESNPCIQNNSCAGLEQEPMVSCKFNMTGLKRDKKKEYNETWYSRDLVCEQSTNESESKCYMNHCNTSVIQESCD

FCo70v1 ............................................~~...S..E..........K...................................................................

FCo70v2 ............................................~~.....K.......D..M...............................................G....................

FCo70v3 ............................................~~.....K..........................................................G....................

FCo70v4 ............................................~~.....K.......D..M....................................................................

FCo70v5 ....................................-.......~~.....K.......D..M...............................................G....................

FCo70v6 ............................................~~.....K.......D..M....................................................................

FCo70v7 ............................................~~...S..E..........K...................................................................

FCo70v8 .................I.............E...........A~~.K...................................................P..........G....................

FCo70v9 ....V.......................................~~.....K.......D..M....................................................................

FCo70v10 ............................................~~.....K.......D..M....................................................................

FCo70v11 ............................................~~.....K.......D..M....................................................................

FCo70v12 ............................................~~.....K.......D..M.........................G.....................................-....

FCo70v13 ............................................~~.....K.......D..M...................................................................N

FCo70v14 ............................................~~.....K.......D..M....................................................................

FCo70v15 ........................................GT.P~~.....K...........K....E..............................................................

FCo70v16 ............................................~~.....K.......D..M....................................................................

FCo70v17 ............................................~~.....K.......D..M..................S.................................................

FCo70v18 ............................................~~...S..E..........K....E..............................................................

FCo70v19 ............................................~~.....K.......D..M....................................................................

FCo70v20 ............................................~~.....K.......D..M.....................................K.........G....................

FCo70v21 ............................................~~.....K.......D..M...............................................G....................

FCo70v22 ............................................~~.....K.......D..M...............................................G....................

FCo70v23 ............................................~~.....K.......D..M...............................................G.....H..............

**SM3 Day 70**

10 20 30 40 50 60 70 80 90 100 110 120

QAIEDVWNLFETSIKPCVKLTPLCIAMRCNKTETDKWGLTRNAT~~TTPDVTAKVINESNPCIQNNSCAGLEQEPMVSCKFNMTGLKRDKKKEYNETWYSRDLVCEQSTNESESKCYMNHCNTSVIQESCD

FGu70v1 .............................S.A...........A~~.K..............................................................GN...................

FGu70v2 ............................................~~...S.............K....E..............................................................

FGu70v3 .........L..................................~~...S.............K....E..............................................................

FGu70v4 ...................................R....G...~~.................K....E.......................................A......................

FGu70v5 ............................................~~.K...A.......D..M....................................................................

FGu70v6 .........................T..............GT..~~.V..E..............S.................................................................

FGu70v7 ............................................~~.K..............................................................G....................

FGu70v8 .............................S.A...........A~~.K...................................................................................

FGu70v9 .............................S.A...........A~~.K...................................................................................

FGu70v10 ...................................R.......A~~.................K....E..............................................................

FGu70v11 .....................................R..GT.P~~.................K....E..............................................................

FGu70v12 .....................................R..GT.P~~.................E.S.................................................................

FGu70v14 .....................................R..GT.P~~.................K....E..............................................................

FGu70v15 .........................T..............GT..~~.A..E........S...R.S.................................................................

FGu70v16 .............................S.A...........A~~.K...........................................R..................GN...................

FGu70v17 ...............................E...........A~~.K..............................................................G...................G

**RM1 Day 70**

10 20 30 40 50 60 70 80 90 100 110 120

QAIEDVWNLFETSIKPCVKLTPLCIAMRCNKTETDKWGLTRNAT~~TTPDVTAKVINESNPCIQNNSCAGLEQEPMVSCKFNMTGLKRDKKKEYNETWYSRDLVCEQSTNESESKCYMNHCNTSVIQESCD

RHt70r1 .............................S.A...........A~~.....K.......D..M.......................NK......................GN...................

RHt70r2 ........................T....S.A...........A~~.....K.......D..M.......................N.......................GN...................

RHt70r3 .............................S.A...........A~~.....K.......D..M.......................N.......................GN...................

RHt70r4 .........................T.....A........GT.A~~A...E............R.....................M........................GN...................

RHt70r5 .............................S.A...........A~~.....K.......D..M.......................N.......................GN...................

RHt70r6 .............................S.A...........A~~.....K.......D..M.......................N.......................GN...................

RHt70r7 .............................S.A...........A~~.....K.......D..M.......................N.......................GN...................

RHt70r8 .............................S.A...........A~~.....K.......D..M.......................N.......................GN...................

RHt70r9 .................A...........S.A...........A~~.....K.......D..M.......................N.......................GN...................

RHt70r10 .............................S.A...........A~~.....K.......D..M.......................N.......................GN...................

RHt70r11 .............................S.A...........A~~.....K.......D..M.......................N.......................GN...................

RHt70r12 .........................T.....A........GT.A~~A...E............R.....................M........................GN...................

RHt70r13 ...............................A...........A~~.K...............................T...................................................

RHt70r14 .............................S.A...........A~~.....K.......D..M.......................N.......................GN...................

RHt70r15 .............................S.A...........A~~.....K.......D..M.......................N.......................GN...................

RHt70r16 ...............................E...........A~~KK......................................E.......................GN...................

RHt70r17 .............................S.A...........A~~.....K.......D..M.......................N.......................GN...................

RHt70r18 .............................S.A...........A~~.....K.......D..M.......................N.......................GN...................

RHt70r19 .............................S.A...........A~~.....K.......D..M.......................N.......................GN...................

RHt70v1 .............................S.A...........A~~.K..............................................................GN...................

RHt70v2 .............................S.A...........A~~.....K.......D..M.......................N.......................GN...................

RHt70v3 .............................S.A...........A~~.....K.......D..M.......................N.......................GN...................

RHt70v4 ...............................E...........A~~.K.......V......................................................G....................

RHt70v5 ...............................E...........A~~KK......................................E........................N...................

RHt70v6 .............................S.A...........A~~.K......................................N.......................GN...................

RHt70v7 .............................S.A...........A~~.K......................................N.......................GN...................

RHt70v8 .............................S.A...........A~~.K......................................N.......................GN...................

RHt70v9 .............................S.A...........A~~.....K.......D..M.......................N.......................GN...................

RHt70v10 .............................S.A...........A~~.....K.......D..M.......................N.......................GN...................

RHt70v11 .............................S.A...........A~~.....K.......D..M.......................N.......................GN...................

RHt70v12 .............................S.A...........A~~.....K.......D..M.......................N.......................GN...................

RHt70v13 .............................S.A...........A~~.....K.......D..M.......................N.......................GN...................

RHt70v14 ...............................E...........A~~KK......................................E...R...................GN...................

RHt70v15 ............................R..E...........A~~KK......................................E.......................GN...................

RHt70v16 .............................S.A...........A~~.K.......................................K......................GN...................

RHt70v17 .............................S.A...........A~~.....K.......D..M.......................N.......................GN...................

RHt70v18 .............................S.A...........A~~.....K.......D..M.......................N........V...........G..GN...................

RHt70v19 .............................S.A...........A~~.....K.......D..M.......................N.......................GN...................

RHt70v20 ...............................A...........A~~.K...............................T...................................................

RHt70v21 .............................S.A...........A~~.....K.......D..M.......................N.......................GN...................

**RM3 Day 70**

10 20 30 40 50 60 70 80 90 100 110 120

QAIEDVWNLFETSIKPCVKLTPLCIAMRCNKTETDKWGLTRNAT~~TTPDVTAKVINESNPCIQNNSCAGLEQEPMVSCKFNMTGLKRDKKKEYNETWYSRDLVCEQSTNESESKCYMNHCNTSVIQESCD

RZw70v2 .............................S.....R....GT..~~AA..E..............S.....................K......................GN...................

RZw70v3 .............................S.A...........A~~.K..............................................................G....................

RZw70v4 .............................S.....R....GT..~~AA..E..............S.....................K......................GN...........C.......

RZw70v5 .............................S.....R....GT..~~AA..E..............S.....................K......................GN...................

RZw70v6 .............................S.....R....GT..~~AA..E..............S.....K...............K......................GN........R..........

RZw70v7 .............................D.A.A.........A~~.K.................S............................................G....................

RZw70v8 .............................S.....R....GT..~~AA..E..............S.........V...........K..................RG..GN.....R...H.........

RZw70v9 .............................S.....R....GT..~~AA..E..............S.....K...............K...................KS.GN........R..........

RZw70v10 .............................D.A.A.........A~~.K.................S............................................G....................

RZw70v11 .............................D.A.A.........A~~.K..............................................................G....................

RZw70v12 .............................S.....R....GT..~~AA..E..............S.....................K......................GN...................

RZw70v13 .............................S.....R....GT..~~AA..E..............S.....K...............K......................GN...................

RZw70v14 .............................S.....R....GT..~~AA..E..............S.....................K......................GN...................

RZw70v15 .............................S...A.......T.A~~.K..............................................................G....................

**SM1 Day 100**

10 20 30 40 50 60 70 80 90 100 110 120

QAIEDVWNLFETSIKPCVKLTPLCIAMRCNKTETDKWGLTRNAT~~TTPDVTAKVINESNPCIQNNSCAGLEQEPMVSCKFNMTGLKRDKKKEYNETWYSRDLVCEQSTNESESKCYMNHCNTSVIQESCD

FLn100v1 ............................................~~.....A.......D..M....................................................................

FLn100v2 ............................................~~.....K.......D..M....................................................................

FLn100v3 ............................................~~.....A.......D..M....................................................................

FLn100v4 ............................................~~.....A.......D..M...................................C................................

FLn100v5 ............................................~~.....A.......D..M....................................................................

FLn100v6 ..T.........................................~~.....A.......D..M....................................................................

FLn100v7 ............................................~~.....A.......D..M.................................................K..................

FLn100v8 ............................................~~.....A.......D..M....................................................................

FLn100v9 ............................................~~.....A.......D..M....................................................................

FLn100v10 .........................................Y..~~.....A.......D..M.........................G..........................................

FLn100v11 ............................................~~.....K.......D..M....................................................................

FLn100v12 ....Y........................D.A.AE........A~~.K..........R...................................................G....................

FLn100v14 ......R......T...............D.A.A.........A~~.K..............................................................G....................

**SM2 Day 100**

10 20 30 40 50 60 70 80 90 100 110 120

QAIEDVWNLFETSIKPCVKLTPLCIAMRCNKTETDKWGLTRNAT~~TTPDVTAKVINESNPCIQNNSCAGLEQEPMVSCKFNMTGLKRDKKKEYNETWYSRDLVCEQSTNESESKCYMNHCNTSVIQESCD

FCo100v1 .....................................R..GT.S~~.................K....E.........R...................C................................

FCo100v2 .....................................R..GT.P~~.................K....E....................................................S.........

FCo100v3 .....................................R..GT.P~~.................K....E..............................................................

FCo100v4 ...............................E...........A~~.K..............................YT................................................P..

FCo100v5 .....................................R..GT.P~~.................K....E..............................................................

FCo100v6 ................R....................R..GT.P~~.................K....E..............................................................

FCo100v7 .............................D.A...........A~~.K..............................................................G.................F..

FCo100v8 .....................................R..GT.P~~.................K....E..............................................................

FCo100v9 .....................................R..GT.P~~.................K....E..............................................................

FCo100v10 .............................D.A.A.........A~~.K..............................................................G....................

FCo100v11 .....................................R..GT.P~~.................K....E..............................................................

FCo100v12 ..................E..................R..GT.P~~.................K....E..............................................................

FCo100v13 .....................................R..GT.P~~.................KS...E..............................................................

FCo100v14 .............................D.A.A.........A~~.K..............................................................G....................

FCo100v15 .....................................R..GT.P~~.................K....E.................................................S............

FCo100v16 ...................................R........~~.....K.......D.YM................................................G...................

FCo100v17 .....................................R..GT.P~~.................K....E..............................................................

FCo100v18 ...........A................R........R..GT.P~~.................K....E..............................................................

**SM3 Day 100**

10 20 30 40 50 60 70 80 90 100 110 120

QAIEDVWNLFETSIKPCVKLTPLCIAMRCNKTETDKWGLTRNAT~~TTPDVTAKVINESNPCIQNNSCAGLEQEPMVSCKFNMTGLKRDKKKEYNETWYSRDLVCEQSTNESESKCYMNHCNTSVIQESCD

FGu100v1 .............T.......................R..GT.P~~.................K....E..............................................................

FGu100v2 .............................D.A.A.........A~~.K..............................................................G....................

FGu100v3 ..............................R......R..GT.P~~.................K....E............................................................R.

FGu100v4 ..........K..........................R..GT.P~~.................K....E..............................................................

FGu100v5 .............................D.A.A.........A~~.K..............................................................G................G...

FGu100v6 ...................................R........~~.....K.......D.YM....................................................................

FGu100v7 .....................................R..GT.P~~........A........K....E............D..............................................P..

FGu100v8 .............................D.A.A.........A~~.K..............................................................G....................

FGu100v9 .............................D.A.A.........A~~.K..............................................................G....................

FGu100v11 ................R............D.A.A.........A~~.K..............................................................G...R................

FGu100v12 ..........K..........................R..GT.P~~.................-....E..............................................................

FGu100v14 ...............................E...........A~~.K.........G.....R..............YT...................................................

FGu100v15 .....................................R..GT.L~~.................K....E..............................................................

**RM1 Day 100**

10 20 30 40 50 60 70 80 90 100 110 120

QAIEDVWNLFETSIKPCVKLTPLCIAMRCNKTETDKWGLTRNAT~~TTPDVTAKVINESNPCIQNNSCAGLEQEPMVSCKFNMTGLKRDKKKEYNETWYSRDLVCEQSTNESESKCYMNHCNTSVIQESCD

RHt100r1. .............................S.A...........A~~.....K.......D..M........................K......................GN.....K.............

RHt100r2 ...............................E...........A~~.K......................................N.......................GN...................

RHt100r3 .............................S.A...........A~~.....K.......D..M.......................N.......................GN...................

RHt100r4 .............................S.A...........A~~.....K.......D..M........................K......................GN...................

RHt100r5 ...............................E...........A~~.KS.....................................N.......................GN...................

RHt100r6 ..................R..........S.A...........A~~.K........D.............................N.......................GN...................

RHt100r7 ...................................E.......A~~.K...K.......D.....S............................S....................................

RHt100r8 ...............................E.........D.A~~KK......................................E.......................GN...................

RHt100r9 ...................................E.......A~~.K...K.......D.....S............................S....................................

RHt100v9 ...................................E.......A~~.K...K.......D.....S............................S....................................

RHt100r10 ...................................E.......A~~.K...K.......D.....S............................S....................................

RHt100r12 .............................S.A...........A~~.K................D.....................N.......................GN...............G...

RHt100r13 ...............................A...E.......A~~.K...K.......D.....S............................S....................................

RHt100r15 .............................S.A...........A~~.....K.......D..M........................K......................GN...................

RHt100r16 .............................S.A...........A~~.K........D.............................N.......................GN...................

RHt100r17 ..V..........................S.A...........A~~.K........D.............................N.......................GN...................

RHt100r18 ...........................G...E.....R..GT.P~~.................K....E..............................................................

**RM3 Day 100**

10 20 30 40 50 60 70 80 90 100 110 120

QAIEDVWNLFETSIKPCVKLTPLCIAMRCNKTETDKWGLTRNAT~~TTPDVTAKVINESNPCIQNNSCAGLEQEPMVSCKFNMTGLKRDKKKEYNETWYSRDLVCEQSTNESESKCYMNHCNTSVIQESCD

RZw100v1 ........................................GT.P~~.................K....E..............................................................

RZw100v2 .............................D.A.A.........A~~.K..................................L...........................G........R...........

RZw100v3 .....................................R..GT.P~~.................K....E............................................R.................

RZw100v4 .....................................R..GT.P~~.................K....E..............................................................

RZw100v5 .....................................R..GT.P~~.................K....E..............................................................

RZw100v6 .....................................R..GT.P~~.................K....E..............................................................

RZw100v7 .....................................R..GT.P~~.................K....E................................................I.............

RZw100v8 ....................I................RI.GT.P~~........A.............E..............................................................

RZw100v9 .....................................R.AGT.P~~.................K....E..................K...........................................

RZw100v10 ..................E..........D.A.A.........A~~.K.......V......................................................G...............H....

RZw100v11 .....................................R..GT.P~~.................K....E..............................................................

RZw100v12 .....................................R..GA.P~~.................K....E..............................................................

RZw100v13 .....................................R..GT.P~~.................K....E....................R.........................................

RZw100v14 .............................D.A.A.........A~~.K..............................................................G....................

RZw100v15 ......S.....................................~~..TTTPDV.....D..M....................................................................

RZW100v16 ............P...............................~~.....A.......D..M.............I......................................................

RZw100v17 .............................D.A.A.........A~~.K..............................................................G....................

RZw100v18 ............................................~~..TTTPDV.....D..M....................................................................

**SM1 Day 578**

10 20 30 40 50 60 70 80 90 100 110 120

QAIEDVWNLFETSIKPCVKLTPLCIAMRCNKTETDKWGLTRNAT~~TTPDVTAKVINESNPCIQNNSCAGLEQEPMVSCKFNMTGLKRDKKKEYNETWYSRDLVCEQSTNESESKCYMNHCNTSVIQESCD

262FLn578 ............................................~~.....A.......A..M....................................................................

241FLn578 ...................................R........~~...N..E......................................-......................E................

243FLn578 ...................................R........~~...N..E..............................................................................

247FLn578 .........................T.........Q....GT..~~I...........ND.....S.................................................................

248FLn578 ...................................R.....DT.~~.A...........A..M.......................................................S............

250FLn578 ..........................................T.~~.....A.......A..M......................................G.............................

252FLn578 ..........................................T.~~.....A.......A..M....................................................................

253FLn578 .........................T.........Q....GT.A~~A...E.....K..D..M....................................................................

254FLn578 ....E....................T.........Q....GT.A~~A...E.....K..D..M...................................................................G

255FLn578 ...................................R.....DT.~~.A...........A..M.......................................................S............

257FLn578 ............................................~~............RD..L...............................................K......I.............

260FLn578 ...................................R........~~...N..E..............................................................................

261FLn578 .........................T.........Q....GT.A~~A...E.....K..D..M....................................................................

240FLn578 ..........................................T.~~.....A...V...A..M....................................................................

**SM2 Day 578**

10 20 30 40 50 60 70 80 90 100 110 120

QAIEDVWNLFETSIKPCVKLTPLCIAMRCNKTETDKWGLTRNAT~~TTPDVTAKVINESNPCIQNNSCAGLEQEPMVSCKFNMTGLKRDKKKEYNETWYSRDLVCEQSTNESESKCYMNHCNTSVIQESCD

491FCo578 ....H...................................GT..~~.............D..M...............................................G....................

376FCo578 .....................H............G.....GT.P~~.K...............K....E.........................................G..G.................

377FCo578 ..V.....................................GT.P~~................................................................G....................

379FCo578 ....N.....K.............................GT.P~~.........................K......…......G......................K...........S........

482FCo578 ........................................GT.P~~...N.K..A........K....E.........................................G....................

483FCo578 .........S..............................GT.P~~...N.K...........K....E......................................G.......................

484FCo578 ........................................GT.P~~...N.K...........K...RE..............................................................

485FCo578 ........................................GT.P~~...N.K...........K...RE..............................................................

487FCo578 ........................................GT.P~~A....K...........K....E............................-......G..........................

489FCo578 ........................................GT.P~~A....N...........K....E...........................................G..................

490FCo578 ........................................GT.P~~.......................................................................V.............

375FCo578 ..............E.........................GT.P~~................................................................G....................

**SM3 Day 578**

10 20 30 40 50 60 70 80 90 100 110 120

QAIEDVWNLFETSIKPCVKLTPLCIAMRCNKTETDKWGLTRNAT~~TTPDVTAKVINESNPCIQNNSCAGLEQEPMVSCKFNMTGLKRDKKKEYNETWYSRDLVCEQSTNESESKCYMNHCNTSVIQESCD

407FGu578 .........................................D..~~...R..........L..K....E..............................................................

190FGu578 .........................T...........R..GT.P~~...N.............KD...E..............................................................

191FGu578 .....................................R..GT.A~~...N......D......K....E..............................................................

386FGu578 ........................................GT..~~.A...K....D..YL..KD...E..............................................................

388FGu578 .........................T..............GT..~~.....K.R.....DS..K.S.........................R.......................................

389FGu578 .........................T..............GT..~~.A...K.......YL..K....E..............................................................

390FGu578 .........................T..............GT..~~.A...KP......YL..K....E..............................................................

391FGu578 .........................T..............GT..~~.A...K.......YL..K....ES.............................................................

393FGu578 .........................T..............GT..~~.A...K.......YS..K....E....V.........................................................

394FGu578 .........................T..............GT..~~.A...K.......YL..K....E.......................K......................................

396FGu578 ...........................................A~~...N......S......K....E.......................................A......................

397FGu578 ....................N...................GT..~~.A...K.......YL..K....E.....................R........................................

399FGu578 ......................I..T..............GT..~~.P...K.....G.YL..K....E..............................................................

400FGu578 .........................TT.............GT..~~.A...K.......YL..K....E..............................................................

401FGu578 .................................A......GT..~~.A...K.......YL..K....E..............................................................

405FGu578 .........................T..............GT..~~A...EA.......DLR.K....E......................................N.T.....................

189FGu578 .....................................R..GT.P~~...N.............KD...E...........L...........................A......................

**RM1 Day 578**

10 20 30 40 50 60 70 80 90 100 110 120

QAIEDVWNLFETSIKPCVKLTPLCIAMRCNKTETDKWGLTRNAT~~TTPDVTAKVINESNPCIQNNSCAGLEQEPMVSCKFNMTGLKRDKKKEYNETWYSRDLVCEQSTNESESKCYMNHCNTSVIQESCD

502RHt578 ................S...N...................GT..~~.A...K.......YL..K....E.........................S............N........C..............

273RHt578 .....................................R..GT.P~~.................K....E..............................................................

278RHt578 ..............................R.............~~A..NE.......GS..........................N.......................G....................

282RHt578 .............................S.....R....GT..~~.A..E........S...R.S..............................A..................................

493RHt578 .........................T..............GT..~~.A...K.......YL..K....E.........................S............N....G...C.....S........

494RHt578 ............F............T...............S..~~...R.........Y...K....E.....................R........................................

496RHt578 .........................T.............AGT..~~.A...K.......YL..K....E.........................S....................................

497RHt578 .........................T..............GT..~~.A...K.......YL..K....E.........................S............N........C..............

498RHt578 ....................N...................GT..~~.A...K.......YL..K....E............D........R........................................

499RHt578 .........................T...............D..~~.A...K.......YL..K....E.....................R........................................

500RHt578 ....................N...................GT..~~.A...K.......YL..K....E.....................R........................................

501RHt578 .........................T...............S..~~...R.........Y...K.D..E..................G..R........................................

270RHt578 ..............R.....S.......................~~A..TE..R..D.............................N.....................A.G....................

**RM3 Day 578**

10 20 30 40 50 60 70 80 90 100 110 120

QAIEDVWNLFETSIKPCVKLTPLCIAMRCNKTETDKWGLTRNAT~~TTPDVTAKVINESNPCIQNNSCAGLEQEPMVSCKFNMTGLKRDKKKEYNETWYSRDLVCEQSTNESESKCYMNHCNTSVIQESCD

565RZw578 ...............................A........GS..~~A..S.........D..M.....P..G....I.........N............................................

506RZw578 ....................N...................GT..~~.A...K.......YL..K....E.....................R........................................

507RZw578 ....................N..........A........GT..~~.A...K.......YL..K....E.....................R...S....................................

513RZw578 ...................SN...................GT..~~.A...K.......YL..K....E.....................R........................................

544RZw578 ............................................~~.....K.......D..T....................................................................

545RZw578 ............................................~~.....K......RD..L...............................................K......I.............

546RZw578 ....N.......................R........R..GT.P~~...N.........D...K....E..............A...............................................

547RZw578 ....Y.......................................~~.....K.......D..T....................................................................

548RZw578 ............................................~~.....K.......D..T....................................................................

549RZw578 HT..........................................~~A...........RD..L...............................................K......I.............

551RZw578 ............................................~~.....K.......D..T..............................................................T.....

552RZw578 ..........................L..........R..GT.P~~...N.............K....E.........................D....................................

553RZw578 ............................................~~.....K.......D..T..............................................................T.....

554RZw578 ...................................R.....DT.~~.A...........A..M.......................................................S............

555RZw578 ............................................~~.....K.......D..T....................................................................

556RZw578 .................A..........................~~..S..........D..M.....P..G....I.........N.......................GN...................

558RZw578 ............................................~~...G........RD..L...............................................K......I.............

559RZw578 ........................................G...~~A..S.........D..M.....P..G....I.........N............................................

560RZw578 ............................................~~..S..........D..M.....P..G....I.........N.......................GN...................

561RZw578 ..............E.........................G...~~A..S.........D..M.....P..G....I.........N.........................G..................

562RZw578 ............................R........R..GT.P~~...N.........D...K....E.........................................GN...................

564RZw578 ............................................~~...G........RD..L...............................................K......I.............
