# Supplementary material for: SIVsm Quasispecies Adaptation to a New Simian Host
Source: PLoS Pathog. 2005 Sep 30;1(1):e3. doi: 10.1371/journal.ppat.0010003 (PMC1238738; doi:10.1371/journal.ppat.0010003)
Supplement: Figure S6 — Region corresponds to nucleotides 1,516–1,936 of SIVsmmH4. Sequences were aligned using the program CLUSTAL X [41], followed by manual adjustment using MacClade 4.0 [42]. The top sequence in each set corresponds to the majority consensus sequence from all sequences at all time points, with codon positions labeled above. A dot indicates amino acid identity with the consensus sequence, and any amino acid changes are indicated with the appropriate symbol. (75 KB DOC) [file ppat.0010003.sg006.doc]

***SI***

WVKLVEEKKFGAEVVPGFQALSEGCTPYDINQMLNCVGEHQAAMQIIREIINEEAADWDLQHPQPGPIPAGQLRDPRGSDIAGTTSTVEEQIQWMYRQQNPIPVGNIYRRWIQLGLQKCVRMYNPVNILDIKQGPKEPFQ

023FQi ............................................................................................................................................

025FQi .......................A....................................................................................................................

026FQi ............................................................................................................................................

028FQi ...W........................................................................................................................................

029FQi ...........P.............P.....P...................I..............S.........................................................................

030FQi ..........................L.................................................................................................................

032FQi ............................................................................................................................................

***Day 10***

***SM1***

WVKLVEEKKFGAEVVPGFQALSEGCTPYDINQMLNCVGEHQAAMQIIREIINEEAADWDLQHPQPGPIPAGQLRDPRGSDIAGTTSTVEEQIQWMYRQQNPIPVGNIYRRWIQLGLQKCVRMYNPVNILDIKQGPKEPFQ

081FLn10 ......................................G............................................................................................R........

082FLn10 ............................................................................................................................................

083FLn10 ............................................................................................................................................

084FLn10 .........S..................................................................................................................................

085FLn10 ...................T......................................................................................T.................................

086FLn10 ............................................................................................................................................

087FLn10 ............................................................................................................................................

088FLn10 ............................................................................................................................................

090FLn10 ................................................................................................G...........................................

091FLn10 ............................................................................................................................................

***SM2***

WVKLVEEKKFGAEVVPGFQALSEGCTPYDINQMLNCVGEHQAAMQIIREIINEEAADWDLQHPQPGPIPAGQLRDPRGSDIAGTTSTVEEQIQWMYRQQNPIPVGNIYRRWIQLGLQKCVRMYNPVNILDIKQGPKEPFQ

043FCo10 ............................................................................................................................................

044FCo10 .......................................................................................M....................................................

045FCo10 ............................................................................................................................................

046FCo10 .........................P..................................................................................................................

047FCo10 ............................................................................................................................................

048FCo10 ............................................................................................................................................

050FCo10 ............................................................................................................................................

051FCo10 ............................................................................................................................................

***RM1***

WVKLVEEKKFGAEVVPGFQALSEGCTPYDINQMLNCVGEHQAAMQIIREIINEEAADWDLQHPQPGPIPAGQLRDPRGSDIAGTTSTVEEQIQWMYRQQNPIPVGNIYRRWIQLGLQKCVRMYNPVNILDIKQGPKEPFQ

033RHt10 ............................................................................................................................................

034RHt10 ............................................................................................................................................

035RHt10 ............................................................................................................................................

036RHt10 ............................................P...............................................................................................

037RHt10 .................LR.........................................................................................................................

038RHt10 ............................................................................................................................................

039RHt10 ............................................................................................................................................

040RHt10 .....G............................IG.....................................G..................................................................

042RHt10 .......R....................................................................................................................................

***RM2***

WVKLVEEKKFGAEVVPGFQALSEGCTPYDINQMLNCVGEHQAAMQIIREIINEEAADWDLQHPQPGPIPAGQLRDPRGSDIAGTTSTVEEQIQWMYRQQNPIPVGNIYRRWIQLGLQKCVRMYNPVNILDIKQGPKEPFQ

351RQl10 .......................................................................................A....................................................

355RQl10 ............................................................................................................................................

356RQl10 ............................................................................................................................................

357RQl10 ............................................................................................................................................

432RQl10 ....................P.......................................................................................................................

434RQl10 ............................................................................................................................................

435RQl10 ............................................................................................................................................

436RQl10 ............................................................................................................................................

437RQl10 .......................................................................................A....................................................

438RQl10 ............................................................................................................................................

***Day 14***

***SM1***

WVKLVEEKKFGAEVVPGFQALSEGCTPYDINQMLNCVGEHQAAMQIIREIINEEAADWDLQHPQPGPIPAGQLRDPRGSDIAGTTSTVEEQIQWMYRQQNPIPVGNIYRRWIQLGLQKCVRMYNPVNILDIKQGPKEPFQ

057FLn14 ......G.........................................................................T...........................................................

058FLn14 ............................................................................................................................................

059FLn14 ............................................................................................................................................

060FLn14 ....................................................................................A.......................................................

061FLn14 ...............................R..........................................................R.................................................

062FLn14 .......................V....................................................................................................................

063FLn14 ............................................................................................................................................

064FLn14 ............................................................................................................................................

065FLn14 ..............................................T.............................................................................................

066FLn14 ...........................................T.............................G...............D..................................................

***SM2***

WVKLVEEKKFGAEVVPGFQALSEGCTPYDINQMLNCVGEHQAAMQIIREIINEEAADWDLQHPQPGPIPAGQLRDPRGSDIAGTTSTVEEQIQWMYRQQNPIPVGNIYRRWIQLGLQKCVRMYNPVNILDIKQGPKEPFQ

057FLn14 ......G.........................................................................T...........................................................

144FCo14 .............................T.....Y........................................................................................................

145FCo14 ............................................................................................................................................

146FCo14 .......................................................................................................................................R....

147FCo14 ............................................................................................................................................

148FCo14 ............................................................................................................................................

149FCo14 ............................................................................................................................................

151FCo14 ............................................................................................................................................

153FCo14 ............................................................................................................................................

154FCo14 ............................................................................................................................................

***SM3***

WVKLVEEKKFGAEVVPGFQALSEGCTPYDINQMLNCVGEHQAAMQIIREIINEEAADWDLQHPQPGPIPAGQLRDPRGSDIAGTTSTVEEQIQWMYRQQNPIPVGNIYRRWIQLGLQKCVRMYNPVNILDIKQGPKEPFQ

057FLn14 ......G.........................................................................T...........................................................

055FGu14 ..............................................T...............................................V...................................V.........

056FGu14 ............................................................................................................................................

131FGu14 ............................................................................................................................................

132FGu14 .................................................................................................................................V..........

134FGu14 ............................................................................................................................................

135FGu14 ............................................................................................................................................

136FGu14 ..........................................................................................................................................L.

137FGu14 ...................................................................................I........................................................

138FGu14 ............................................................................................................................................

***RM1***

WVKLVEEKKFGAEVVPGFQALSEGCTPYDINQMLNCVGEHQAAMQIIREIINEEAADWDLQHPQPGPIPAGQLRDPRGSDIAGTTSTVEEQIQWMYRQQNPIPVGNIYRRWIQLGLQKCVRMYNPVNILDIKQGPKEPFQ

057FLn14 ......G.........................................................................T...........................................................

141RHt14 ............................................................................................................................................

450RHt14 ............................................................................................................................................

451RHt14 ............................................................................................................................................

452RHt14 ............................................................................................................................................

453RHt14 ........................................R......................................................................T............................

455RHt14 ............................................................................................................................................

456RHt14 .....................................................................V......................................................................

458RHt14 .....G......................................................................................................................................

461RHt14 ..............A.............................................................................................................................

462RHt14 ............................................................................................................................................

***RM2***

WVKLVEEKKFGAEVVPGFQALSEGCTPYDINQMLNCVGEHQAAMQIIREIINEEAADWDLQHPQPGPIPAGQLRDPRGSDIAGTTSTVEEQIQWMYRQQNPIPVGNIYRRWIQLGLQKCVRMYNPVNILDIKQGPKEPFQ

057FLn14 ......G.........................................................................T...........................................................

408RQl14 ...............................................................................................................R...........................

409RQl14 ............................................................................................................................................

410RQl14 ............................................................................................................................................

411RQl14 ......................................................................................................................................T.....

412RQl14 .....G......................................................................................................................................

413RQl14 ............................................................................................................................................

415RQl14 ............................................................................................................................................

416RQl14 ...............................R............................................................................................................

417RQl14 ................................V...........................................................................................................

418RQl14 ............................................................................................................................................

419RQl14 ............................................................................................................................................

420RQl14 ............................................................................................................................................

421RQl14 ............................................................................................................................................

422RQl14 ............................................................................................................................................

423RQl14 ............................................................................................................................................

424RQl14 ............................................................................................................................................

425RQl14 ............................................................................................................................................

***RM3***

WVKLVEEKKFGAEVVPGFQALSEGCTPYDINQMLNCVGEHQAAMQIIREIINEEAADWDLQHPQPGPIPAGQLRDPRGSDIAGTTSTVEEQIQWMYRQQNPIPVGNIYRRWIQLGLQKCVRMYNPVNILDIKQGPKEPFQ

057FLn14 ......G.........................................................................T...........................................................

094RZw14 ............................................................................................................................................

095RZw14 ...........................................T........................................A........................................A..............

096RZw14 ............................................................................................................................................

097RZw14 ..R.............................................G......T.................................................S..................................

099RZw14 .L....K............P.........................F..................................T...........................................................

100RZw14 ............................................................................................................................................

101RZw14 .........................................................................................................D..................................

102RZw14 ......................................................P.....................................................................................

103RZw14 ............................................................................................................................................

104RZw14 ....L......................N................................................................................................................

217RZw14 ............................................................................................................................................

218RZw14 ..................................................................................E.........................................................

219RZw14 ................................................V.................................A.....................................................Q...

233RZw14 .................................................T..........................................................................................

238RZw14 ............................................................................................................................................

***Day 100***

***SM1***

WVKLVEEKKFGAEVVPGFQALSEGCTPYDINQMLNCVGEHQAAMQIIREIINEEAADWDLQHPQPGPIPAGQLRDPRGSDIAGTTSTVEEQIQWMYRQQNPIPVGNIYRRWIQLGLQKCVRMYNPVNILDIKQGPKEPFQ

220FLn100 ............................................................................................................................................

221FLn100 ..................................D.........................................................................................................

222FLn100 .........................................................................................................................V..................

223FLn100 ............................................................................................................................................

224FLn100 .........................................V..................................................................................................

225FLn100 ............................................................................................................................................

226FLn100 ..............................................V.............................................................................................

228FLn100 ................................................................................T...........................................................

229FLn100 ............................................................................................................................................

231FLn100 .......................................R....................................................................................................

232FLn100 ............................................................................................................................................

***SM2***

WVKLVEEKKFGAEVVPGFQALSEGCTPYDINQMLNCVGEHQAAMQIIREIINEEAADWDLQHPQPGPIPAGQLRDPRGSDIAGTTSTVEEQIQWMYRQQNPIPVGNIYRRWIQLGLQKCVRMYNPVNILDIKQGPKEPFQ

195FCo100 ............................................................................................................................................

196FCo100 ............................................................................................................................................

198FCo100 ................................................................................T...........................................................

199FCo100 ............................................................................................................................................

200FCo100 ............................................................................................................................................

201FCo100 ....L............................................................S..........................................................................

202FCo100 ............................................................................................................................................

***SM3***

WVKLVEEKKFGAEVVPGFQALSEGCTPYDINQMLNCVGEHQAAMQIIREIINEEAADWDLQHPQPGPIPAGQLRDPRGSDIAGTTSTVEEQIQWMYRQQNPIPVGNIYRRWIQLGLQKCVRMYNPVNILDIKQGPKEPFQ

204FGu100 ..................................................V.........................................................................................

206FGu100 ............................................................................................................................................

208FGu100 ...............................................................................N............................................................

209FGu100 ............................................................................................................................................

211FGu100 ............................................................................................................................................

213FGu100 ............................................................................................................................................

215FGu100 ............................................................................................................................................

***RM1***

WVKLVEEKKFGAEVVPGFQALSEGCTPYDINQMLNCVGEHQAAMQIIREIINEEAADWDLQHPQPGPIPAGQLRDPRGSDIAGTTSTVEEQIQWMYRQQNPIPVGNIYRRWIQLGLQKCVRMYNPVNILDIKQGPKEPFQ

070RHt100 ......................................D.....................................................................................................

073RHt100 ......................................D.....................................................................................................

075RHt100 ......................................D.....................................................................................T...............

076RHt100 ......................................D.....................................................................................................

077RHt100 ......................................D.....................................................................................................

078RHt100 ......................................D.....................................................................................................

079RHt100 ................................................................................T...........................................................

***RM3***

WVKLVEEKKFGAEVVPGFQALSEGCTPYDINQMLNCVGEHQAAMQIIREIINEEAADWDLQHPQPGPIPAGQLRDPRGSDIAGTTSTVEEQIQWMYRQQNPIPVGNIYRRWIQLGLQKCVRMYNPVNILDIKQGPKEPFQ

362RZw100 ............................................................................................................................................

367RZw100 ...........T...............................................................................T................................................

368RZw100 ............................................................................................................................................

370RZw100 ..........................................................................................R..........M.........T............................

374RZw100 ............................................................................................................................................

***Day 578***

***SM1***

WVKLVEEKKFGAEVVPGFQALSEGCTPYDINQMLNCVGEHQAAMQIIREIINEEAADWDLQHPQPGPIPAGQLRDPRGSDIAGTTSTVEEQIQWMYRQQNPIPVGNIYRRWIQLGLQKCVRMYNPVNILDIKQGPKEPFQ

308FLn578 ....................................A.......................................................................................................

309FLn578 ............................................................................................................................................

310FLn578 ............................................................................................................................................

313FLn578 .................S.....................R...........................................A........................................................

314FLn578 .................S.....................R...........................................A........................................................

315FLn578 ............................................................................................................................................

***SM2***

WVKLVEEKKFGAEVVPGFQALSEGCTPYDINQMLNCVGEHQAAMQIIREIINEEAADWDLQHPQPGPIPAGQLRDPRGSDIAGTTSTVEEQIQWMYRQQNPIPVGNIYRRWIQLGLQKCVRMYNPVNILDIKQGPKEPFQ

463FCo578 ............................................................................................................................................

464FCo578 ............................................................................................................................................

465FCo578 R............................................................................................................................I..............

466FCo578 .................L...........................................................................................................I..............

467FCo578 .......................................................................................A....................................................

468FCo578 ............................................................................................................................................

469FCo578 ............................................................................................................................................

470FCo578 ............................................................................................................................................

471FCo578 .............................................................................................................................I..............

472FCo578 .............................................................................................................................I..............

473FCo578 ............................................R...............................................................................................

474FCo578 ...............................................................................................................T.............I..............

475FCo578 ............................................R...............................................................................................

476FCo578 .............................................................................................................................I..............

477FCo578 ............................................................................................................................................

478FCo578 ..........................................................................................R.................................................

***SM3***

WVKLVEEKKFGAEVVPGFQALSEGCTPYDINQMLNCVGEHQAAMQIIREIINEEAADWDLQHPQPGPIPAGQLRDPRGSDIAGTTSTVEEQIQWMYRQQNPIPVGNIYRRWIQLGLQKCVRMYNPVNILDIKQGPKEPFQ

175FGu578 ............................................................................................................................................

179FGu578 ............................................................................................................................................

181FGu578 ..................................................................................................L.........................................

184FGu578 ............................................................................................................................................

429FGu578 .............................................................................................................................A..............

430FGu578 .....................................................................................................................................E......

431FGu578 ............................................................................................................................................

***RM1***

WVKLVEEKKFGAEVVPGFQALSEGCTPYDINQMLNCVGEHQAAMQIIREIINEEAADWDLQHPQPGPIPAGQLRDPRGSDIAGTTSTVEEQIQWMYRQQNPIPVGNIYRRWIQLGLQKCVRMYNPVNILDIKQGPKEPFQ

330RHt578 ......................................D............................V........................................................................

331RHt578 ...............................E......D.E..........................V........................................................................

333RHt578 ...................................R..D............................V........................................................................

336RHt578 ......................................D.........................L..V........................................................................

342RHt578 ......................................D........G...................V........................................................................

343RHt578 ......................................D................................................................................A....................

345RHt578 ......................................D...........................SV........................................................................

346RHt578 ...........T..........................D............................V.....................................D.......................G..........

347RHt578 ......................................D............................V........................................................................

348RHt578 ......................................D............................V........................................................................

349RHt578 ......................................D............................V........................................................................

***RM3***

WVKLVEEKKFGAEVVPGFQALSEGCTPYDINQMLNCVGEHQAAMQIIREIINEEAADWDLQHPQPGPIPAGQLRDPRGSDIAGTTSTVEEQIQWMYRQQNPIPVGNIYRRWIQLGLQKCVRMYNPVNILDIKQGPKEPFQ

441RZw578 ....................................I..............................V........................................................................

443RZw578 ....................................I..............................V........................................................................

446RZw578 ...................................................................V........................................................................

447RZw578 ...................................................................V.......................................H...V............................

448RZw578 ...................................................................V.......................................H...V............................

449RZw578 ....................................I..............................V........................................................................
